# Three Decades of Adolescent Health: Unveiling Global Trends Across 41 Countries in Psychological and Somatic Complaints (1994–2022)

**DOI:** 10.3389/ijph.2024.1607774

**Published:** 2024-12-02

**Authors:** Karen Schrijvers, Alina Cosma, Thomas Potrebny, Einar Thorsteinsson, Carolina Catunda, Franziska Reiss, Sabina Hulbert, Michaela Kostičová, Marina Melkumova, Michela Bersia, Helena Jeriček Klanšček, Tania Gaspar, Maxim Dierckens

**Affiliations:** ^1^ Department of Public Health and Primary Care, Ghent University, Ghent, Belgium; ^2^ School of Psychology, Trinity College Dublin, Dublin, Ireland; ^3^ Olomouc University Social Health Institute, Palacky University Olomouc, Olomouc, Czechia; ^4^ Department of Health and Functioning, Western Norway University of Applied Sciences, Bergen, Norway; ^5^ School of Psychology, University of New England, Armidale, NSW, Australia; ^6^ Department of Social Sciences, University of Luxembourg, Esch-sur-Alzette, Luxembourg; ^7^ Department of Child and Adolescent Psychiatry, Psychotherapy, and Psychosomatics, University Medical Center Hamburg-Eppendorf, Hamburg, Germany; ^8^ Centre for Health Services Studies (CHSS), University of Kent, Canterbury, United Kingdom; ^9^ Institute of Social Medicine and Medical Ethics, Faculty of Medicine, Comenius University in Bratislava, Bratislava, Slovakia; ^10^ Arabkir Medical Centre, Institute of Child and Adolescent Health, Yerevan, Armenia; ^11^ Department of Public Health and Pediatrics, University of Torino, Torino, Italy; ^12^ National Institute of Public Health, Ljubljana, Slovenia; ^13^ School of Psychology and Life Sciences, SPIC/Hei-Lab/Lusófona University, Lisbon, Portugal

**Keywords:** adolescence, mental health, gender differences, cross-national, HBSC

## Abstract

**Objectives:**

This study examined (non-)monotonic time trends in psychological and somatic complaints among adolescents, along with gender differences.

**Methods:**

Repeated cross-sectional Health Behaviour in School-aged Children (HBSC) data from 1994 to 2022 covering 15-year-old adolescents from 41 countries (N = 470,797) were analysed. Three polynomial logistic regression models (linear, quadratic, cubic) were tested for best fit, including separate analyses by gender and health complaints dimension.

**Results:**

Time trend patterns varied by gender and health complaints dimension. Increases were found in 82.3% of cases (linear 25%, quadratic U-shaped 28.7%, cubic 28.7%), while 14% showed no clear trend, and 3.7% decreased. Boys typically showed linear increases or no clear trend over time, whereas girls generally showed cubic or U-shaped trends. Psychological complaints often displayed U-shaped or cubic patterns, whereas somatic complaints mostly showed linear increases.

**Conclusion:**

Psychological and somatic complaints demonstrated diverse time trend patterns across countries, with non-monotonic patterns (U-shaped and cubic) frequently observed alongside linear increases. These findings highlight the complexity of changes within countries over three decades, suggesting that linear modelling may not effectively capture this heterogeneity.

## Introduction

Good health and wellbeing underpin crucial developmental tasks associated with adolescent years [1], of which mental health is a fundamental component. However, more than one-third of adolescents often experience a variety of psychological and somatic complaints [2, 3]. These complaints serve as important indicators of difficulties in mental health and wellbeing and can be stress reactions to psychosocial tensions in adolescents’ lives without a clear organic cause [4, 5]. Moreover, psychological and somatic complaints can negatively affect adolescents’ general health, functioning, and wellbeing, potentially leading to mental health issues later in life [6] that significantly contribute to the global disease burden [7]. Recent evidence has indicated an increase in psychological and somatic complaints over time [8], possibly influenced by national-level events and cross-national crises like the 2008 economic crisis and the COVID-19 pandemic as well as increased mental health awareness and availability of mental health services. Long-term time trend analyses are essential to fully understand these changes and guide efforts to monitor and improve the overall wellbeing of adolescents.

The four-yearly cross-national Health Behaviour in School-aged Children (HBSC) study provides a unique opportunity to monitor time trends in adolescent health and wellbeing using the HBSC Multiple Health Complaints scale. Initially introduced in 1986, the scale was refined in 1994 to measure eight items, encompassing somatic (headache, backache, stomachache, and dizziness) and psychological (feeling nervous, feeling low, feeling irritable, and difficulties in getting to sleep) complaints. Since then, the scale has remained unchanged [9]. Somatic and psychological complaints tend to be comorbid [10–13] with some researchers treating them as one factor called psychosomatic complaints [13, 14] and others as two different factors (i.e., psychological and somatic complaints) [11, 12, 15, 16]. Both approaches have solid validation evidence and have been used previously in adolescent health research.

Studies utilizing the one-factor approach reported similar trend patterns over time across different national contexts. For example, changes over time in psychosomatic complaints appeared to be relatively stable in the 1990s and early 2000s in countries such as Scotland, England, and the Netherlands [17–19]. However, there has been an increase in the proportion of adolescents reporting two or more psychosomatic complaints from 2010 onwards [17–20]. These results have been further confirmed through pooling of cross-national data [8].

Studies employing the two-factor approach revealed diverse changes over time in psychological and somatic complaints across different national contexts. These studies showed a relatively stable trend for *psychological complaints* during the 1990s and early 2000s, followed by an increase with some differences between countries following the early 2000s. For instance, Switzerland noted a stable trend from 1994 to 2006 [15], while in Norway, an upward trend during the same period was identified, followed by a decrease in 2014 [16]. Recent studies in Czechia (2002–2018), Italy (2010–2018) and Canada (2022–2018, particularly among girls) reported an increase in psychological complaints [20–22]. A recent cross-national study showed a stable trend in psychological complaints between 2002 and 2010 followed by an increase towards 2018 [23], another study using pooled data (2002–2018) reported an increase in psychological complaints over time across the majority of countries [24]. In terms of *somatic complaints*, an increasing trend from the 1990s onwards was noted until the early 2000s in Switzerland, Norway, and Czechia [15, 16, 21], followed by a decrease in Norway (2010–2014) and Czechia (2010–2018) [16, 21]. Conversely, Italy and Canada showed an increase over this same period [20, 22]. An increasing trend in somatic complaints between 2002 and 2018 has also been confirmed in one cross-national study thus far [23]. Given these diverging trends for both psychological and somatic complaints and the lack of studies spanning a long time frame, it is essential to consolidate results in one study and explore both psychological and somatic complaints (i.e., two-factor approach) separately over an extended timeframe and across countries to further elucidate differences in time trends.

Previous studies have mostly employed a monotonic approach and examined linear changes over time in psychosomatic complaints. However, this may not be sufficient to capture the full complexity of these trends [25]. The presence of non-monotonic time trends, such as quadratic (U-shaped or inverted U-shaped) or cubic trends has rarely been examined, except for one study [25]. These non-monotonic time trend patterns are often not considered, although they are important, especially when examining trends over a longer period of time where multiple fluctuations may have occurred. Our study aims to improve the understanding of these developments over a longer period of time and update them with recent representative cross-national data by employing the two-factor approach.

Although divergent trends over time have been reported, some results were also consistent across studies in terms of prevalence rate and gender. Trend studies employing a two-factor approach have generally reported a lower prevalence of somatic compared to psychological complaints [15, 16, 20, 21, 26]. Moreover, a higher prevalence of health complaints, using either the one or two-factor approach, were noted in girls compared to boys [15–21, 26, 27]. Further investigation of the differences in prevalence rates according to gender and health complaints dimension within trend studies considering different trend patterns could provide a more nuanced understanding of these changes over time.

### This Study

The aforementioned studies highlighted the necessity for further investigation into diverse patterns of change over time, beyond the commonly employed linear approach. Therefore, to address these gaps the current study aims to inform policy and signpost key target groups for tailored interventions such as addressing the specific needs of boys and girls in coping with different dimensions of health complaints over time. Moreover, using representative data from adolescents across 41 countries, this study aims to provide insights into potential cross-national disparities, emphasizing the importance of considering diverse cultural and contextual factors alongside gender differences and differences by dimension of health complaints. To gain a deeper understanding of these long-term time trends, the present study spans a period of almost three decades in 41 countries. This research tests which pattern of change over time (i.e., linear, quadratic, cubic) most accurately describes the fluctuations observed over the past three decades in psychological and somatic complaints among adolescents for each country separately. Additionally, it explores whether these different patterns of change over time differ between boys and girls.

## Methods

### Study Design and Sample

Data from the cross-national HBSC study on health and wellbeing in 11-, 13- and 15-year-old adolescents is collected every 4 years. A representative sample is surveyed within the school context in each region/country following the international research protocol [9]. The present study employed data from eight survey cycles, (i.e., 1994, 1998, 2002, 2006, 2010, 2014, 2018, and 2022) spanning three decades. A total of 41 countries were included in the sample, representing 79% of the 52 countries in the dataset. Countries with at least four data points, even if non-consecutive, were included to meet the minimum requirement for testing cubic trends. Systematic previous findings documented that the 15-year-olds report the highest prevalence, the largest gender differences and also the widest cross-national variations [8, 20, 22, 24], therefore this study included only participants from this age group. Our final sample included 470,797 15-year-old adolescents. Sample sizes by country are presented in Supplementary Table S1.

### Measures

Adolescents’ psychological and somatic complaints were measured using the HBSC Multiple Health Complaints scale [9]. Adolescents were asked to indicate how often in the past 6 months they had each of the following eight symptoms: feeling low, irritability or bad temper, feeling nervous, difficulties in getting to sleep, headache, backache, stomachache, and feeling dizzy. Response options ranged from 1 “about every day” to 5 “rarely or never.” A two-dimensional approach was followed with the first four items as a measure for psychological complaints and the last four items indicating somatic complaints [11, 15, 16]. Both variables were dichotomized based on the cut-off of experiencing two or more complaints more than once a week [11, 15, 16, 20]. This cut-off point was used as it was suitable for both psychological and somatic complaints, where a more conservative approach of 3 or more complaints would result in low prevalence rates for somatic complaints and a less conservative approach of 1 or more complaints would result in high prevalence rates for psychological complaints (Supplementary Figure S1). The measure has shown good internal reliability, construct validity [12] and configural and metric cross-national invariance [13]. Gender was measured by asking adolescents whether they are a boy or a girl. Age was determined by asking for the month and year of birth.

### Statistical Analyses

Prevalence rates of psychological and somatic complaints were calculated by country, survey cycle, and gender. To determine the most suitable trend pattern over time within each country, logistic regression analyses were conducted for boys and girls, and for each health complaints dimension, resulting in 164 separate cases (k = 164; 41 countries × 2 genders × 2 dimensions of health complaints). The survey cycle variable (year of data collection) was centred, and orthogonal polynomials were used in the quadratic and cubic models. Three models were tested – including cubic, quadratic, and linear functions – and polynomial regression models were fitted using “glmnet” package in R v4.1-1 (1) [28]. The optimal trend function was determined based on the degree of change and the model significance, employing a conservative p-value criterion (*p* < .001) because of the large sample size [29]. Subsequently, two sensitivity analyses were conducted. First, one on the pooled dataset to evaluate the influence of the selected cut-off point (i.e., at least one, two or three complaints more than once a week) on the results of the trend patterns. Besides, also a sensitivity analysis to assess the COVID-19 impact on the overall trend patterns in adolescents’ psychological and somatic symptoms. This involved rerunning the respective models without the 2022 data and comparing them to the full models with the 2022 data. Sensitivity testing could not be performed for Russia, Ukraine, and the United States due to the unavailability of 2022 data.

## Results

### Descriptives

**FIGURE 1:**
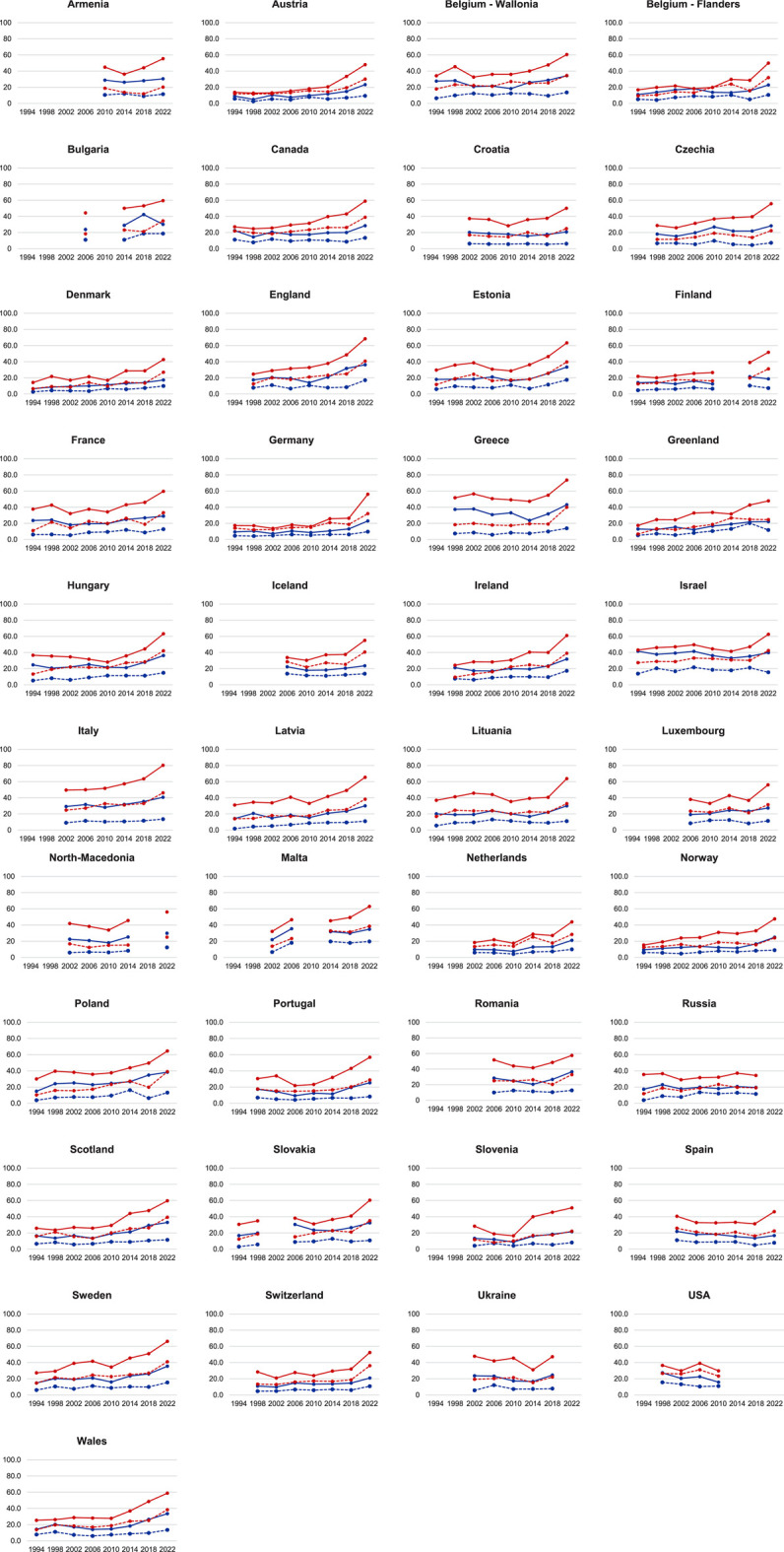
Prevalence of two or more psychological and somatic complaints in boys and girls by country and year (Health Behaviour in School-aged Children study, 1994–2022 for 41 countries). Notes. Solid lines represent prevalence rates for psychological complaints, while dotted lines indicate prevalence rates for somatic complaints. Blue lines represent boys, red lines represent girls.

**FIGURE 2:**
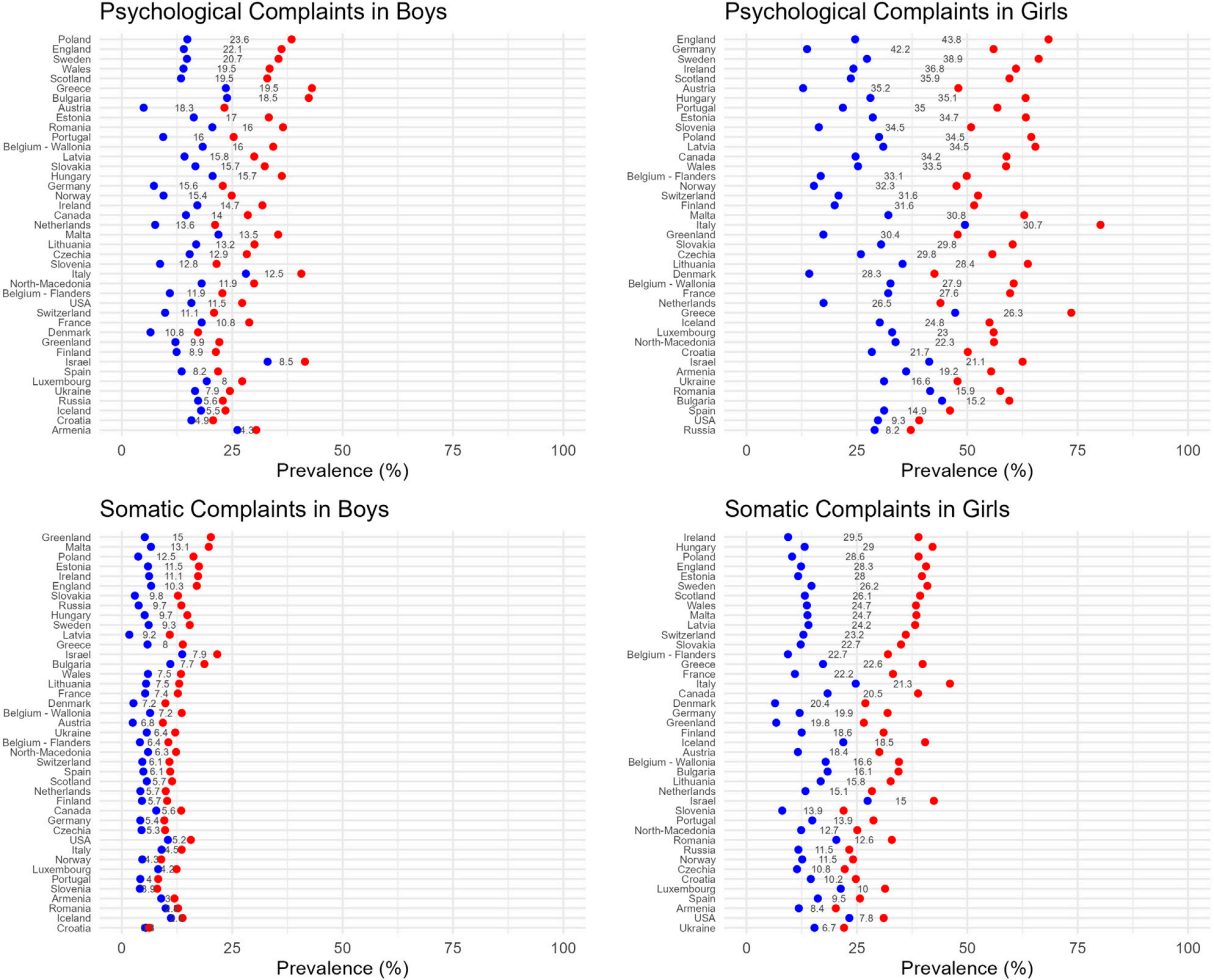
Dumbbell plot showing highest and lowest prevalence rate of two or more psychological and somatic complaints by gender and country across all countries (Health Behaviour in School-aged Children study, 1994–2022 for 41 countries). Notes. The difference between the highest and lowest prevalence is displayed in grey between the dumbbells.

Figure 1 presents the observed prevalence over time by country, while Supplementary Table S2 provides detailed information per survey cycle. Additionally, Figure 2 illustrates the observed gap between the highest and lowest prevalence observed over the study period by country, highlighting a large range of differences over time between countries. Overall, the highest prevalence rates in somatic and psychological complaints were observed in 2022 across most countries for both boys (32 countries) and girls (36 countries). Conversely, there were wide cross-national variations in relation to the lowest prevalence for these indicators over time. More specifically, for psychological complaints, the lowest prevalence within each country could be observed at various points between 1994 and 2014. For somatic complaints within countries the lowest prevalence was found between 1994 and 2018. However, in 1994, data was collected from 20 counties with the prevalence for somatic complaints being the lowest for girls in 17 countries and boys in 13 counties as compared with data collected after 1994.

Across countries, the prevalence rates of psychological complaints were generally higher, ranging from 4.9% in boys from Austria in 1998 to 80.2% in girls from Italy in 2022. In contrast, somatic complaints ranged from 1.7% in boys from Latvia in 1994 to 16.1% in girls from Italy in 2022. Overall, for both psychological and somatic complaints in Italy and Israel high prevalence rates were observed. Specifically for psychological complaints, Greece showed particularly higher prevalence rates. Furthermore, across countries the observed gap over time was generally larger in psychological complaints for both boys and girls, suggesting greater fluctuations over time in psychological complaints compared to somatic complaints. Additionally, girls reported higher levels of psychological and somatic complaints compared to boys across all countries.

### Trend Patterns

Notable cross-national differences in trend patterns for both dimensions of health complaints and genders were found (see Tables 1, 2). Within most countries and for both dimensions of health complaints and genders, 82.3% (*k* = 135) of the observed trend patterns indicated an increase in complaints over the survey period. More specifically, three increasing trend patterns emerged: linear (25%; *k* = 41; i.e., steady increase since the start of the survey), quadratic U-shaped (28.7%; *k* = 47; i.e., an initial decrease followed by an increase in more recent years), and cubic (28.7%; *k* = 47; i.e., multiple fluctuations of increases and decreases over the years with an increase towards the latest datapoint). For a small number of cases (3.7%, *k* = 6) a decrease in psychological and somatic symptoms for boys and girls was found. More specifically, two trend patterns emerged. A linear decrease was observed in 1.8% of cases (*k* = 3), suggesting an overall steady decrease over the years. Additionally, in 1.8% of cases (*k* = 3), an inverted U-shaped trend was identified, indicating an initial increase followed by a subsequent decrease. Finally, in 14% of the cases (*k* = 23) no clear trend pattern was observed, suggesting stability in complaints over the period studied (see also Table 3 for an overview).

**TABLE 1:** Time trend pattern for psychological complaints by country and gender (Health Behaviour in School-aged Children study, 1994–2022 for 41 countries).

	Boys psychological complaints			Girls psychological complaints			Trend pattern	
	Linear trend	Quadratic trend	Cubic trend	Linear trend	Quadratic trend	Cubic trend	Boys	Girls
Armenia	0.002 (0.003)	0.471 (0.451)	−0.195 (0.451)	0.010 (0.002)***	2.311 (0.494)***	−0.751 (0.494)	—	QU
Austria	0.005 (0.000)***	2.242 (0.319)***	0.529 (0.319)	0.013 (0.001)***	5.032 (0.409)***	1.306 (0.409)**	QU	QU
Belgium - Flanders	0.003 (0.000)***	0.803 (0.372)*	2.120.371)***	0.011 (0.001)***	4.273 (0.437)***	2.453 (0.436)***	C	C
Belgium - Wallonia	0.002 (0.001)***	2.990 (0.436)***	0.006 (0.436)	0.008 (0.001)***	3.860 (0.486)***	1.711 (0.486)***	QU	C
Bulgaria	0.007 (0.001)***	−1.231 (0.460)**	−2.541 (0.458)***	0.009 (0.002)***	0.513 (0.497)	0.254 (0.498)	C	LI
Canada	0.002 (0.000)***	2.950 (0.399)***	0.311 (0.399)	0.011 (0.000)***	5.240 (0.471)***	0.692 (0.471)	QU	QU
Croatia	−0.000 (0.001)	0.998 (0.386)*	0.440 (0.386)	0.006 (0.001)***	3.646 (0.479)***	0.437 (0.479)	—	QU
Czechia	0.004 (0.001)***	0.370 (0.419)	0.770 (0.419)	0.012 (0.001)***	2.855 (0.481)***	1.566 (0.481)**	LI	QU
Denmark	0.003 (0.000)***	0.326 (0.313)	0.273 (0.313)	0.008 (0.001)***	2.504 (0.419)***	1.470 (0.419)***	LI	C
England	0.006 (0.001)***	2.318 (0.410)***	0.916 (0.410)*	0.015 (0.001)***	3.503 (0.464)***	1.970 (0.463)***	QU	C
Estonia	0.005 (0.001)***	2.099 (0.411)***	1.398 (0.410)***	0.0090.001)***	4.390 (0.477)***	3.140 (0.475)***	C	C
Finland	0.002 (0.001)***	0.863 (0.357)**	−0.095 (0.357)	0.010 (0.001)***	2.830 (0.439)***	0.660 (0.439)	LI	QU
France	0.003 (0.001)***	2.106 (0.419)***	−0.635 (0.418)	0.007 (0.001)***	4.284 (0.486)***	1.022 (0.486)*	QU	QU
Germany	0.004 (0.000)***	2.199 (0.320)***	1.104 (0.319)***	0.012 (0.001)***	6.287 (0.412)***	2.738 (0.411)***	C	C
Greece	0.001 (0.001)	3.299 (0.473)***	2.001 (0.473)***	0.007 (0.001)***	4.636 (0.488)***	2.966 (0.486)***	C	C
Greenland	0.004 (0.001)***	0.350 (0.365)	−0.213 (0.366)	0.010 (0.001)***	0.236 (0.457)	0.631 (0.457)	LI	LI
Hungary	0.003 (0.001)***	2.337 (0.433)***	0.929 (0.432)*	0.008 (0.001)***	6.123 (0.477)***	2.486 (0.476)***	QU	C
Iceland	0.002 (0.001)*	1.593 (0.400)***	−0.464 (0.400)	0.014 (0.001)***	3.206 (0.479)***	0.510 (0.479)	QU	QU
Ireland	0.003 (0.001)***	1.997 (0.405)***	0.212 (0.405)	0.013 (0.001)***	2.655 (0.467)***	1.072 (0.467)*	QU	QU
Israel	−0.001 (0.001)	1.161 (0.485)**	1.161 (0.485)*	0.005 (0.001)***	3.078 (0.497)***	3.994 (0.495)***	—	C
Italy	0.006 (0.001)***	1.256 (0.469)**	0.302 (0.469)	0.014 (0.001)***	2.857 (0.481)***	0.766 (0.481)	LI	QU
Latvia	0.005 (0.001)***	1.675 (0.403)***	0.938 (0.403)*	0.010 (0.001)***	3.837 (0.482)***	2.360 (0.481)***	QU	C
Lithuania	0.002 (0.001)***	1.485 (0.410)***	1.550 (0.410)***	0.005 (0.001)***	2.998 (0.492)***	4.635 (0.489)***	C	C
Luxembourg	0.005 (0.001)***	0.065 (0.419)	0.156 (0.419)	0.010 (0.001)***	2.436 (0.488)***	0.884 (0.487)	LI	QU
North-Macedonia	0.005 (0.001)***	1.274 (0.421)**	−0.597 (0.421)	0.008 (0.001)***	2.505 (0.490)***	−0.976 (0.490)*	LI	QU
Malta	0.004 (0.002)**	−0.433 (0.460)	1.284 (0.459)**	0.013 (0.002)***	0.768 (0.490)	1.514 (0.489)**	—	LI
Netherlands	0.005 (0.001)***	1.402 (0.326)***	0.181 (0.326)	0.011 (0.001)***	2.221 (0.433)***	0.930 (0.432)*	QU	QU
Norway	0.004 (0.001)***	1.005 (0.336)**	1.253 (0.336)***	0.009 (0.001)***	0.743 (0.431)	1.260 (0.430)**	C	LI
Poland	0.007 (0.001)***	0.859 (0.437)*	1.655 (0.437)***	0.010 (0.001)***	3.432 (0.485)***	2.613 (0.485)***	C	C
Portugal	0.004 (0.001)***	2.708 (0.364)***	0.001 (0.364)	0.011 (0.001)***	5.750 (0.462)***	0.406 (0.462)	QU	QU
Romania	0.006 (0.001)***	2.698 (0.449)***	0.457 (0.449)	0.005 (0.001)***	3.586 (0.497)***	−0.317 (0.497)	QU	QU
Russia	0.000 (0.001)	−0.014 (0.394)	0.289 (0.394)	0.000 (0.001)	1.455 (0.470)**	−0.862 (0.470)	—	—
Scotland	0.006 (0.001)***	2.487 (0.392)***	−0.238 (0.392)	0.012 (0.001)***	4.142 (0.460)***	−0.323 (0.460)	QU	QU
Slovakia	0.004 (0.001)***	0.187 (0.432)	1.815 (0.431)***	0.008 (0.001)***	3.555 (0.480)***	2.722 (0.479)***	C	C
Slovenia	0.005 (0.001)***	1.328 (0.357)***	−0.719 (0.356)*	0.018 (0.001)***	2.746 (0.460)***	−3.616 (0.457)***	QU	C
Spain	−0.003 (0.001)***	0.759 (0.377)*	0.233 (0.377)	0.001 (0.001)	3.419 (0.475)***	0.392 (0.475)	LD	QU
Sweden	0.005 (0.001)***	1.982 (0.411)***	1.562 (0.410)***	0.011 (0.001)***	2.329 (0.485)***	2.273 (0.484)***	C	C
Switzerland	0.003 (0.001)***	0.814 (0.348)*	0.791 (0.348)*	0.009 (0.001)***	4.959 (0.455)***	1.543 (0.455)***	LI	C
Ukraine	−0.001 (0.001)	1.726 (0.408)***	1.235 (0.408)**	−0.003 (0.001)*	2.033 (0.494)***	1.937 (0.493)***	QU	C
USA	−0.008 (0.002)***	0.019 (0.407)	−1.045 (0.407)*	−0.003 (0.002)	−0.150 (0.472)	−2.103 (0.471)***	LD	C
Wales	0.007 (0.000)***	3.682 (0.432)***	1.192 (0.432)**	0.014 (0.001)***	4.918 (0.479)***	0.632 (0.480)	QU	QU

Notes. Estimates (Standard error); P < 0.001 = ***; P < 0.01=**; P < 0.05=*; LI, linear increase; LD, linear decrease; QU, quadratic U-shaped; QIU, quadratic inverted U-shaped; C, cubic.

**TABLE 2:** Time trend pattern for somatic complaints by country and gender (Health Behaviour in School-aged Children study, 1994–2022 for 41 countries).

	Boys somatic complaints			Girls somatic complaints			Trend pattern	
	Linear trend	Quadratic trend	Cubic trend	Linear trend	Quadratic trend	Cubic trend	Boys	Girls
Armenia	0.000 (0.002)	0.224 (0.305)	0.464 (0.305)	0.001 (0.002)	1.671 (0.363)***	0.393 (0.363)	—	QU
Austria	0.001 (0.000)***	0.494 (0.241)*	−0.214 (0.241)	0.006 (0.000)***	2.488 (0.375)***	1.098 (0.375)**	LI	QU
Belgium - Flanders	0.002 (0.000)***	−0.362 (0.271)	0.328 (0.271)	0.007 (0.000)***	1.354 (0.386)***	1.140 (0.386)**	LI	QU
Belgium - Wallonia	0.002 (0.000)**	−0.517 (0.311)	0.800 (0.311)**	0.005 (0.001)***	0.802 (0.430)	1.205 (0.430)**	—	LI
Bulgaria	0.005 (0.001)***	0.634 (0.349)	−0.993 (0.349)**	0.007 (0.001)***	1.247 (0.419)**	1.407 (0.419)***	LI	C
Canada	0.001 (0.000)	0.646 (0.304)*	0.386 (0.304)	0.005 (0.000)***	3.376 (0.432)***	0.847 (0.432)	—	QU
Croatia	0.000 (0.001)	0.116 (0.234)	0.003 (0.234)	0.003 (0.001)***	1.339 (0.381)***	0.364 (0.381)	—	QU
Czechia	0.000 (0.000)	0.224 (0.243)	0.640 (0.243)**	0.004 (0.001)***	0.544 (0.370)	1.453 (0.370)***	—	C
Denmark	0.002 (0.000)***	0.413 (0.225)	0.210 (0.225)	0.006 (0.000)***	1.443 (0.333)***	1.362 (0.332)***	LI	C
England	0.002 (0.001)***	1.027 (0.293)***	1.075 (0.293)***	0.009 (0.001)***	1.4580.406)***	1.915 (0.405)***	C	C
Estonia	0.003 (0.000)***	0.9910.300)**	1.039 (0.299)***	0.007 (0.001)***	2.279 (0.408)***	3.144 (0.406)***	C	C
Finland	0.001 (0.000)**	−0.188 (0.250)	−0.115 (0.250)	0.005 (0.001)***	1.090 (0.378)**	1.471 (0.378)***	—	C
France	0.002 (0.000)***	0.063 (0.281)	−0.240 (0.281)	0.005 (0.001)***	0.420 (0.403)	1.527 (0.403)***	LI	C
Germany	0.002 (0.000)***	0.391 (0.238)	0.342 (0.238)	0.006 (0.001)***	2.359 (0.380)***	0.614 (0.380)	LI	QU
Greece	0.002 (0.000)***	0.989 (0.287)***	0.352 (0.287)	0.008 (0.001)***	3.825 (0.416)***	2.375 (0.415)***	QU	C
Greenland	0.004 (0.001)***	−0.051 (0.297)	−0.508 (0.296)	0.007 (0.001)***	−0.312 (0.375)	−0.260 (0.375)	LI	LI
Hungary	0.003 (0.000)***	0.073 (0.294)	0.091 (0.294)	0.008 (0.001)***	1.751 (0.422)***	2.104 (0.422)***	LI	C
Iceland	0.000 (0.001)	0.826 (0.328)*	−0.215 (0.328)	0.008 (0.001)***	3.267 (0.446)***	0.346 (0.446)	—	QU
Ireland	0.003 (0.001)***	0.630 (0.295)*	0.537 (0.295)	0.010 (0.001)***	0.787 (0.398)*	1.016 (0.398)*	LI	LI
Israel	3.835 (0.000)	−1.453 (0.384)***	−0.131 (0.384)	0.004 (0.001)***	1.582 (0.469)***	2.291 (0.468)***	QIU	C
Italy	0.002 (0.001)*	0.216 (0.315)	0.387 (0.315)	0.009 (0.001)***	1.067 (0.465)*	1.469 (0.464)**	—	LI
Latvia	0.003 (0.000)***	−0.325 (0.264)	0.063 (0.264)	0.008 (0.001)***	2.419 (0.407)***	1.030 (0.407)*	LI	QU
Lithuania	0.001 (0.000)**	−1.001 (0.298)***	0.753 (0.298)*	0.003 (0.001)***	0.680 (0.422)	2.530 (0.421)***	QIU	C
Luxembourg	0.001 (0.001)	−0.355 (0.304)	0.848 (0.304)**	0.004 (0.001)**	0.944 (0.433)*	0.768 (0.433)	—	—
North-Macedonia	0.003 (0.001)***	0.486 (0.269)	0.054 (0.269)	0.005 (0.001)***	1.666 (0.372)***	−0.277 (0.372)	LI	LI
Malta	0.005 (0.001)***	−0.845 (0.369)*	0.761 (0.368)*	0.011 (0.001)***	−0.482 (0.447)	0.660 (0.446)	LI	LI
Netherlands	0.002 (0.001)***	0.644 (0.249)*	−0.044 (0.249)	0.006 (0.001)***	0.567 (0.390)	0.348 (0.390)	LI	LI
Norway	0.001 (0.000)**	0.261 (0.248)	−0.287 (0.248)	0.003 (0.001)***	0.398 (0.364)	0.478 (0.364)	—	LI
Poland	0.003 (0.000)***	−0.441 (0.282)	0.202 (0.282)	0.008 (0.001)***	1.520 (0.404)***	1.654 (0.404)***	LI	C
Portugal	0.001 (0.000)*	0.548 (0.241)*	−0.212 (0.241)	0.005 (0.001)***	2.420 (0.389)***	0.531 (0.389)	—	QU
Romania	0.001 (0.001)	0.016 (0.321)	0.662 (0.321)*	0.004 (0.001)***	1.691 (0.442)***	1.542 (0.441)***	—	C
Russia	0.003 (0.001)***	−1.171 (0.303)***	−0.193 (0.303)	0.003 (0.001)***	−1.039 (0.384)**	−0.461 (0.384)	QIU	LI
Scotland	0.002 (0.000)***	0.473 (0.275)	−0.103 (0.275)	0.006 (0.001)***	3.095 (0.403)***	0.701 (0.403)	LI	QU
Slovakia	0.003 (0.000)***	−0.828 (0.288)**	−0.119 (0.288)	0.007 (0.001)***	1.656 (0.405)***	1.061 (0.405)**	LI	QU
Slovenia	0.001 (0.001)*	0.252 (0.237)	0.410 (0.237)	0.007 (0.001)***	0.912 (0.355)**	−0.843 (0.354)**	LI	LI
Spain	−0.002 (0.001)**	0.136 (0.278)	−0.063 (0.278)	−0.002 (0.001)***	1.299 (0.406)**	−0.178 (0.406)	—	LI
Sweden	0.002 (0.000)***	0.436 (0.299)	0.904 (0.299)**	0.006 (0.001)***	1.442 (0.428)***	2.007 (0.427)***	LI	C
Switzerland	0.002 (0.000)***	0.492 (0.248)*	0.624 (0.248)**	0.007 (0.001)***	2.913 (0.389)***	2.231 (0.388)***	LI	C
Ukraine	0.000 (0.001)	−0.477 (0.272)	1.025 (0.272)***	0.000 (0.001)	0.344 (0.399)	1.170 (0.398)**	C	—
USA	−0.004 (0.001)**	0.385 (0.331)	0.218 (0.331)	−0.002 (0.002)	−0.901 (0.441)*	−1.140 (0.440)**	—	—
Wales	0.002 (0.000)***	1.622 (0.309)***	0.649 (0.309)*	0.009 (0.000)***	3.797 (0.444)***	2.401 (0.443)***	QU	C

Notes. Estimates (Standard error); P < 0.001 = ***; P < 0.01=**; P < 0.05=*; LI, linear increase; LD, linear decrease; QU, quadratic U-shaped; QIU, quadratic inverted U-shaped; C = cubic.

**TABLE 3:** Overview of time trend patterns for psychological and somatic complaints by gender and country for 1994–2018 and 1994–2022 (Health Behaviour in School-aged Children study, 41 countries).

	Trend until 2018				Trend until 2022			
	Psychological complaints		Somatic complaints		Psychological complaints		Somatic complaints	
Country/region	Boys	Girls	Boys	Girls	Boys	Girls	Boys	Girls
Armenia^e^	—	—	—	Linear decrease	—	Quadratic—U-shaped*	—	Quadratic—U-shaped*
Austria^a^	Linear increase	Quadratic—U-shaped	—	Linear increase	Quadratic—U-shaped*	Quadratic—U-shaped	Linear increase*	Quadratic—U-shaped*
Belgium—Flanders^a^	—	Linear increase	Quadratic—inverted U-shaped	Cubic	Cubic*	Cubic*	Linear increase*	Quadratic—U-shaped*
Belgium—Wallonia^a^	Quadratic—U-shaped	Quadratic—U-shaped	Quadratic—inverted U-shaped	Linear increase	Quadratic—U-shaped	Cubic*	—*	Linear increase
Bulgaria^d^	Quadratic—U-shaped	Linear increase	Quadratic—U-shaped	—	Cubic*	Linear increase	Linear increase*	Cubic*
Canada^a^	—	Quadratic—U-shaped	—	Linear increase	Quadratic—U-shaped*	Quadratic—U-shaped	—	Quadratic—U-shaped*
Croatia^c^	—	Quadratic—U-shaped	—	—	—	Quadratic—U-shaped	—	Quadratic—U-shaped*
Czechia	Linear increase	Linear increase	—	Quadratic—inverted U-shaped	Linear increase	Quadratic—U-shaped*	—	Cubic*
Denmark^a^	Linear increase	Linear increase	Linear increase	Linear increase	Linear increase	Cubic*	Linear increase	Cubic*
England^b^	Cubic	Linear increase	—	Linear increase	Quadratic—U-shaped*	Cubic*	Cubic*	Cubic*
Estonia^a^	—	Cubic	—	Cubic	Cubic*	Cubic	Cubic*	Cubic
Finland^a^	—	Quadratic—U-shaped	Linear increase	Linear increase	Linear increase*	Quadratic—U-shaped	—*	Cubic*
France^a^	Quadratic—U-shaped	Quadratic—U-shaped	Cubic	Quadratic—inverted U-shaped	Quadratic—U-shaped	Quadratic—U-shaped	Linear increase*	Cubic*
Germany^a^	—	Quadratic—U-shaped	—	Linear increase	Cubic*	Cubic*	Linear increase*	Quadratic—U-shaped*
Greece^b^	Linear decrease	Cubic	—	—	Cubic*	Cubic	Quadratic—U-shaped*	Cubic*
Greenland^a^	—	Linear increase	Linear increase	Linear increase	Linear increase*	Linear increase	Linear increase	Linear increase
Hungary^a^	—	Cubic	Linear increase	Linear increase	Quadratic—U-shaped*	Cubic	Linear increase	Cubic*
Iceland^d^	—	Linear increase	—	Cubic	Quadratic—U-shaped*	Quadratic—U-shaped*	/	Quadratic—U-shaped*
Ireland^b^	—	Linear increase	—	Linear increase	Quadratic—U-shaped*	Quadratic—U-shaped*	Linear increase*	Linear increase
Israel^a^	Linear decrease	—	—	—	—*	Cubic*	Quadratic–inverted U-shaped*	Cubic*
Italy^c^	—	Linear increase	—	Linear increase	Linear increase*	Quadratic—U-shaped*	/	Linear increase
Latvia^a^	Linear increase	Linear increase	Linear increase	Linear increase	Quadratic—U-shaped*	Cubic*	Linear increase	Quadratic—U-shaped*
Lithuania^a^	—	Cubic	Quadratic - inverted U-shaped	—	Cubic*	Cubic	Quadratic-inverted U-shaped	Cubic*
Luxembourg^d^	—	Cubic	—	—	Linear increase*	Quadratic—U-shaped*	—	—
North-Macedonia^c^	—	Quadratic—U-shaped	—	—	Linear increase*	Quadratic—U-shaped	Linear increase*	Linear increase*
Malta^c^	—	Linear increase	Linear increase	Linear increase	—	Linear increase	Linear increase	Linear increase
Netherlands^c^	—	Linear increase	—	Cubic	Quadratic—U-shaped*	Quadratic—U-shaped*	Linear increase*	Linear increase*
Norway^a^	—	Linear increase	—	—	Cubic*	Linear increase	—	Linear increase*
Poland^a^	Cubic	Cubic	Quadratic—inverted U-shaped	Linear increase	Cubic	Cubic	Linear increase*	Cubic*
Portugal^b^	Quadratic—U-shaped	Quadratic—U-shaped	—	—	Quadratic—U-shaped	Quadratic—U-shaped	/	Quadratic—U-shaped*
Romania^d^	—/	Quadratic - U-shaped	—	—	Quadratic—U-shaped*	Quadratic—U-shaped	/	Cubic*
Russia^a^	—	—	Quadratic—inverted U-shaped	Linear increase	—	—	—	—
Scotland^a^	Quadratic—U-shaped	Quadratic—U-shaped	—	Quadratic—U-shaped	Quadratic—U-shaped	Quadratic—U-shaped	Linear increase*	Quadratic—U-shaped
Slovakia^a^	Linear increase	—	Linear increase	Linear increase	Cubic*	Cubic*	Linear increase	Quadratic—U-shaped*
Slovenia^c^	Quadratic—U-shaped	Cubic	—	Linear increase	Quadratic—U-shaped	Cubic	Linear increase*	Linear increase
Spain^c^	Linear decrease	Linear decrease	—	Linear decrease	Linear decrease	Quadratic—U-shaped*	—	Linear decrease
Sweden^a^	Linear increase	Linear increase	—	Linear increase	Cubic*	Cubic*	Linear increase*	Cubic*
Switzerland^b^	—	Quadratic—U-shaped	—	Linear increase	Linear increase*	Cubic*	Linear increase*	Cubic*
Ukraine^c^	Quadratic—U-shaped	Cubic	Cubic	—	—	—	—	—
USA^b^	Linear decrease	Cubic	—	—	—	—	—	—
Wales^a^	Cubic	Quadratic—U-shaped	—	Linear increase	Quadratic—U-shaped*	Quadratic—U-shaped	Quadratic—U-shaped*	Cubic*
*Total number of countries with*								
Linear decrease	4	1	0	2	1	0	0	1
Linear increase	6	14	7	20	8	4	19	9
Quadratic—inverted U-shaped	0	0	5	2	0	0	2	0
Quadratic—U-shaped	7	13	1	1	15	18	2	11
Cubic	3	9	2	4	10	16	2	16
None (—)	21	4	26	12	4	0	13	1

* = Different trend pattern for 1994–2018 and 1994–2022;

^a^ Data since 1994.

^b^ Data since 1998.

^c^ Data since 2002.

^d^ Data since 2006.

^e^ Data since 2010.

The findings also indicated several differences in the pattern of change for boys and girls. In general, linear increases (32.9%, k = 27) over time or no clear trend patterns (23.2%, k = 19) were more common among boys, whereas cubic (41.5%, k = 34) or U-shaped (35.4%, k = 29) trends were more common among girls. Therefore, there were greater fluctuations in trends over time among girls compared to boys within the countries included in this study. Furthermore, the results revealed notable differences in trend patterns by *health complaints dimensions*. The most common patterns observed for psychological complaints were U-shaped (41.5%, k = 34) and cubic (34.1%, k = 28) time trends, while linear increases (35.4%, k = 29) were the predominant pattern for somatic complaints, followed by both cubic (23.2%, k = 19) and stable (20.7%, k = 17) time trends. Specifically, for *boys* non-monotonic trends were observed in *psychological symptoms* with U-shaped trends seen in 15 countries and cubic trends in 10 countries. In 8 countries a linear increase was found. Conversely, *somatic complaints* among *boys* predominantly exhibited linear increases in 19 countries, with U-shaped and cubic trends being less prevalent, observed in 2 and 3 countries respectively. In the case of *girls*, U-shaped trends were most common in *psychological complaints* across 18 countries, while cubic trends were prominent in 16 countries, with linear increases noted in 4 countries. Similarly, for *somatic complaints among girls*, cubic trends prevailed in 16 countries, followed by U-shaped trends in 11 countries, with linear increases observed in 10 countries.

### Sensitivity Testing

Sensitivity checks on the cut-off point of at least one, two or three complaints more than once a week on the pooled dataset showed similar conclusions for each selected cut-off for both boys and girls and both health complaints dimensions (Supplementary Table S3).

As COVID-19 may have had a considerable impact on the latest data [30], sensitivity testing was performed without the 2022 data (Supplementary Tables S4, S5). Table 3 illustrates an overview of the findings from the trend pattern testing, including 2022 data, and the sensitivity testing. After excluding the 2022 data, the initially observed trend patterns changed in several countries, likely showing the impact of COVID-19 on the overall time trends.

For *psychological complaints* among the 38 countries included in the sensitivity testing, the trend pattern changed in 26 countries for boys and in 18 countries for girls. For *boys*, the most common impact of 2022 data was that several countries showed no clear trend pattern until 2018, whereas by including the 2022 data this changed to a linear increase (*k* = 6), a U-shaped trend (*k* = 6), or a cubic trend (*k* = 5). For *girls*, the most common impact of the 2022 data encountered, was that the existing linear increase until 2018, became a more complex U-shaped (*k* = 5) or cubic trend (*k* = 5).

Similarly, concerning *somatic complaints*, sensitivity analyses showed that the trend pattern changed in 20 countries for boys and in 28 countries among girls when the 2022 data were included, as opposed to when it was excluded. In the case of somatic complaints in *boys*, the most notable impact of including the 2022 data was that linear increases emerged (*k* = 9), whereas without the 2022 data, often no clear trend was observed., Among *girls*, similarly to psychological complaints, the most common change contains a linear increase observed until 2018, whereas when including the 2022 data, a cubic trend (*k* = 9) was found. In addition, five countries that showed no clear trend pattern until 2018, shifted to a cubic trend when 2022 data were included.

## Discussion

Our study – spanning almost three decades (from 1994 to 2022) and covering 41 countries – aimed to identify the most accurate patterns of change over time (i.e., linear, cubic, and quadratic) in psychological and somatic complaints, as well as explore gender differences therein among representative samples of 15-year-old adolescents.

Within most countries an increase in both psychological and somatic complaints was observed since 1994 with either a steady increasing trend (i.e., linear trend), a decrease followed by an increase (i.e., U-shaped trend), or a fluctuating trend with an increase towards 2022 (i.e., cubic trend). Only within a small number of countries stability or a decrease in psychological and somatic complaints over time was observed. Furthermore, our analyses revealed that the trend patterns among girls exhibited greater complexity, characterised by a higher prevalence of U-shaped and cubic patterns, in contrast to boys, where many patterns remained linear, particularly in somatic complaints. These more intricate patterns suggest heightened fluctuations over time. This might be linked to school stress, body image issues, obesity, and bullying which have been shown to disproportionately affect girls [24, 31].

In comparison to previous research [25], which used data from 1994 to 2010, a shift in trend patterns was observed. While Ottová-Jordan et al. [25] indicated a relatively even distribution between stable, increasing (linear or U-shaped), or decreasing trend patterns (linear, inverted-U-shaped), with only four countries exhibiting an unsteady trend pattern (i.e., cubic), the current study revealed a notable shift in this distribution, with increases becoming the predominant trend pattern by early 2020s. In general, the inclusion of 2022 data demonstrated the acceleration of this upward trend in recent years with non-monotonic time trends becoming more prevalent, which has also been confirmed by previous studies. This may be attributed to the impact of the COVID-19 pandemic, however, the limited number of decreasing time trends in 2018 and a higher number of increasing time trends compared to Ottová-Jordan [25] suggests that this concerning upwards trend was already established prior to the pandemic. Therefore, it is imperative to closely monitor future trends to provide insight into whether COVID-19 has temporarily increased the prevalence or whether the alarming time trend has continued.

In addition, considerable variations in trend patterns for psychological and somatic complaints were observed, with psychological complaints often characterised by complex fluctuations over time, in comparison to the more stable trajectory of somatic complaints. Moreover, numerous cross-national variations in the observed trend patterns by gender and health complaints dimensions were identified. However, no discernible clusters of countries with similar trend patterns were observed. These results indicate that a linear approach in many countries may not be sufficient to capture the complexities of trends over three decades, particularly in girls and in psychological complaints.

Previous research has also well-established a higher prevalence of psychological and somatic complaints among girls compared to boys, along with more pronounced increases over time in girls, which may be reflected in the more complex trend patterns observed in girls compared to boys [32, 33]. Moreover, some countries showed particularly high prevalence rates, like Israel and Italy for both Dimensions of health complaints, with Greece showing higher psychological complaints. Structural factors and macro-level changes may partially explain these findings. For instance, in Israel, political and economic instability and violence exposure contribute to adolescents’ mental health issues [34, 35]. In Greece, the refugee crisis and economic hardship worsen health inequities and mental health problems among adolescents [36, 37]. In Italy, the severe COVID-19 impact and prolonged school closures, along with increased school pressure and social media influence, have led to high psychosomatic complaints in adolescents [38, 39].

The sensitivity test confirmed that for both boys and girls the patterns became more complex when 2022 data (i.e., post-COVID period) were added. For boys in many countries there was stability until 2018, which changed to an upward trend pattern when 2022 data were included. For girls most of the time, existing linear increases became more complex trend patterns with 2022 data included. This sensitivity test illustrated an acceleration of a pre-existing upward trend for girls, whereas for boys it illustrated the beginning of an increase.

In addition to the observed rise in psychological and somatic complaints, potentially attributable to COVID-19, there have been multiple notable fluctuations over time. While this study did not address these fluctuations in detail, it is plausible that various cross-national crises and societal developments have contributed to the observed rise in adolescent psychological and somatic complaints. For example, past crises such as the 2008 economic recession have been associated with adverse effects on adolescent mental health [40]. Additionally, factors such as climate change [41, 42], and armed conflicts [35, 43], may have contributed to the increase in psychosomatic complaints in recent years.

Beyond crises, societal developments have played an important role, with both positive and negative effects on adolescent mental health. Developments such as the widespread availability of the internet since the 1990s and the emergence of social media in the mid-2000s are likely to have negatively influenced adolescent mental health [24, 44], although some show no impact or a positive one [45, 46]. Furthermore, increased awareness of mental health issues, coupled with greater availability of mental health services and increased prevention efforts, may have positively influenced mental health outcomes [47–51]. Also, individual-level factors and specific events at the national level may have influenced these time trends in psychological and somatic complaints as well. All these factors, and probably many more, are likely to have contributed to the observed variations in trend patterns. However, this was not the primary objective of this study, and it is therefore uncertain whether the observed fluctuations in adolescent psychological and somatic complaints can be attributed to these factors.

### Strengths, Limitations and Future Research

The strength of the present study was the use of repeated cross-sectional data from the HBSC study in a large sample of countries, which has adhered to a standardised protocol over time. This provided a unique opportunity to examine 30-year trend patterns across multiple countries, enriching our understanding of the dynamics of adolescent health and wellbeing. However, some limitations need to be considered. The rapid expansion of the HBSC network over the past two decades, led to inconsistent data availability across countries, with some countries lacking data starting from 1994, thereby limiting the examination of trend patterns. Additionally, reliance on self-report measures introduces potential bias, as increased awareness of mental health issues may lead to more reported symptoms, aligning with the prevalence inflation hypothesis [50]. Also, although psychological and somatic complaints are more pronounced in 15-year-olds, studying a wider age range in future studies might provide a better overview of these time trends and their development. Furthermore, the different time trend patterns observed for psychological and somatic complaints in most countries raised questions about the relationship between these two dimensions. Future research is needed to investigate whether there might be a different relationship between the two dimensions over time. Future studies could also focus clusters of countries and include macro-level indicators such as cultural factors or characteristics of welfare and healthcare systems to examine these matters in greater depth. Lastly, the nature of the Multiple Health Complaints Scale, which primarily measures internalizing complaints, could bias the results regarding gender differences. From a developmental perspective, girls are generally more prone to internalizing complaints, whereas boys are more prone to externalizing complaints [5]. Therefore, internalising instruments might not fully capture psychological distress in boys and results should be interpreted with caution.

### Conclusion

In conclusion, an upward trend in psychological and somatic complaints has been observed cross-nationally in recent years. Furthermore, non-monotonical trend patterns have been observed more frequently in girls compared to boys and in psychological compared to somatic complaints. The most prevalent patterns were U-shaped and cubic patterns, in addition to linear increases. However, the identified trend patterns included many cross-national variations. The diverse trend patterns highlight the complexity of the time trends and suggest that a linear approach might be insufficient for capturing the full scope of these trends over three decades.
