# Corrigendum: Three Decades of Adolescent Health: Unveiling Global Trends Across 41 Countries in Psychological and Somatic Complaints (1994–2022)

**DOI:** 10.3389/ijph.2025.1609116

**Published:** 2025-10-23

**Authors:** Karen Schrijvers, Alina Cosma, Thomas Potrebny, Einar Thorsteinsson, Carolina Catunda, Franziska Reiss, Sabina Hulbert, Michaela Kostičová, Marina Melkumova, Michela Bersia, Helena Jeriček Klanšček, Tania Gaspar, Maxim Dierckens

**Affiliations:** ^1^ Department of Public Health and Primary Care, Ghent University, Ghent, Belgium; ^2^ School of Psychology, Trinity College Dublin, Dublin, Ireland; ^3^ Olomouc University Social Health Institute, Palacky University Olomouc, Olomouc, Czechia; ^4^ Department of Health and Functioning, Western Norway University of Applied Sciences, Bergen, Norway; ^5^ School of Psychology, University of New England, Armidale, NSW, Australia; ^6^ Department of Social Sciences, University of Luxembourg, Esch-sur-Alzette, Luxembourg; ^7^ Department of Child and Adolescent Psychiatry, Psychotherapy, and Psychosomatics, University Medical Center Hamburg-Eppendorf, Hamburg, Germany; ^8^ Centre for Health Services Studies (CHSS), University of Kent, Canterbury, United Kingdom; ^9^ Institute of Social Medicine and Medical Ethics, Faculty of Medicine, Comenius University in Bratislava, Bratislava, Slovakia; ^10^ Arabkir Medical Centre, Institute of Child and Adolescent Health, Yerevan, Armenia; ^11^ Department of Public Health and Pediatrics, University of Torino, Torino, Italy; ^12^ National Institute of Public Health, Ljubljana, Slovenia; ^13^ School of Psychology and Life Sciences, SPIC/Hei-Lab/Lusófona University, Lisbon, Portugal

**Keywords:** adolescence, mental health, gender differences, cross-national, HBSC

There was a mistake in Table 3 as published. The headers in the first two rows in table three were off. The corrected Table 3 appears below.

**TABLE 3:** Overview of time trend patterns for psychological and somatic complaints by gender and country for 1994–2018 and 1994–2022 (Health Behaviour in School-aged Children study, 41 countries).

	Trend until 2018				Trend until 2022			
	Psychological complaints		Somatic complaints		Psychological complaints		Somatic complaints	
Country/region	Boys	Girls	Boys	Girls	Boys	Girls	Boys	Girls
Armenia^e^	—	—	—	Linear decrease	—	Quadratic—U-shaped*	—	Quadratic—U-shaped*
Austria^a^	Linear increase	Quadratic—U-shaped	—	Linear increase	Quadratic—U-shaped*	Quadratic—U-shaped	Linear increase*	Quadratic—U-shaped*
Belgium—Flanders^a^	—	Linear increase	Quadratic—inverted U-shaped	Cubic	Cubic*	Cubic*	Linear increase*	Quadratic—U-shaped*
Belgium—Wallonia^a^	Quadratic—U-shaped	Quadratic—U-shaped	Quadratic—inverted U-shaped	Linear increase	Quadratic—U-shaped	Cubic*	—*	Linear increase
Bulgaria^d^	Quadratic—U-shaped	Linear increase	Quadratic—U-shaped	—	Cubic*	Linear increase	Linear increase*	Cubic*
Canada^a^	—	Quadratic—U-shaped	—	Linear increase	Quadratic—U-shaped*	Quadratic—U-shaped	—	Quadratic—U-shaped*
Croatia^c^	—	Quadratic—U-shaped	—	—	—	Quadratic—U-shaped	—	Quadratic—U-shaped*
Czechia	Linear increase	Linear increase	—	Quadratic—inverted U-shaped	Linear increase	Quadratic—U-shaped*	—	Cubic*
Denmark^a^	Linear increase	Linear increase	Linear increase	Linear increase	Linear increase	Cubic*	Linear increase	Cubic*
England^b^	Cubic	Linear increase	—	Linear increase	Quadratic—U-shaped*	Cubic*	Cubic*	Cubic*
Estonia^a^	—	Cubic	—	Cubic	Cubic*	Cubic	Cubic*	Cubic
Finland^a^	—	Quadratic—U-shaped	Linear increase	Linear increase	Linear increase*	Quadratic—U-shaped	—*	Cubic*
France^a^	Quadratic—U-shaped	Quadratic—U-shaped	Cubic	Quadratic—inverted U-shaped	Quadratic—U-shaped	Quadratic—U-shaped	Linear increase*	Cubic*
Germany^a^	—	Quadratic—U-shaped	—	Linear increase	Cubic*	Cubic*	Linear increase*	Quadratic—U-shaped*
Greece^b^	Linear decrease	Cubic	—	—	Cubic*	Cubic	Quadratic—U-shaped*	Cubic*
Greenland^a^	—	Linear increase	Linear increase	Linear increase	Linear increase*	Linear increase	Linear increase	Linear increase
Hungary^a^	—	Cubic	Linear increase	Linear increase	Quadratic—U-shaped*	Cubic	Linear increase	Cubic*
Iceland^d^	—	Linear increase	—	Cubic	Quadratic—U-shaped*	Quadratic—U-shaped*	/	Quadratic—U-shaped*
Ireland^b^	—	Linear increase	—	Linear increase	Quadratic—U-shaped*	Quadratic—U-shaped*	Linear increase*	Linear increase
Israel^a^	Linear decrease	—	—	—	—*	Cubic*	Quadratic–inverted U-shaped*	Cubic*
Italy^c^	—	Linear increase	—	Linear increase	Linear increase*	Quadratic—U-shaped*	/	Linear increase
Latvia^a^	Linear increase	Linear increase	Linear increase	Linear increase	Quadratic—U-shaped*	Cubic*	Linear increase	Quadratic—U-shaped*
Lithuania^a^	—	Cubic	Quadratic - inverted U-shaped	—	Cubic*	Cubic	Quadratic-inverted U-shaped	Cubic*
Luxembourg^d^	—	Cubic	—	—	Linear increase*	Quadratic—U-shaped*	—	—
North-Macedonia^c^	—	Quadratic—U-shaped	—	—	Linear increase*	Quadratic—U-shaped	Linear increase*	Linear increase*
Malta^c^	—	Linear increase	Linear increase	Linear increase	—	Linear increase	Linear increase	Linear increase
Netherlands^c^	—	Linear increase	—	Cubic	Quadratic—U-shaped*	Quadratic—U-shaped*	Linear increase*	Linear increase*
Norway^a^	—	Linear increase	—	—	Cubic*	Linear increase	—	Linear increase*
Poland^a^	Cubic	Cubic	Quadratic—inverted U-shaped	Linear increase	Cubic	Cubic	Linear increase*	Cubic*
Portugal^b^	Quadratic—U-shaped	Quadratic—U-shaped	—	—	Quadratic—U-shaped	Quadratic—U-shaped	/	Quadratic—U-shaped*
Romania^d^	—/	Quadratic - U-shaped	—	—	Quadratic—U-shaped*	Quadratic—U-shaped	/	Cubic*
Russia^a^	—	—	Quadratic—inverted U-shaped	Linear increase	—	—	—	—
Scotland^a^	Quadratic—U-shaped	Quadratic—U-shaped	—	Quadratic—U-shaped	Quadratic—U-shaped	Quadratic—U-shaped	Linear increase*	Quadratic—U-shaped
Slovakia^a^	Linear increase	—	Linear increase	Linear increase	Cubic*	Cubic*	Linear increase	Quadratic—U-shaped*
Slovenia^c^	Quadratic—U-shaped	Cubic	—	Linear increase	Quadratic—U-shaped	Cubic	Linear increase*	Linear increase
Spain^c^	Linear decrease	Linear decrease	—	Linear decrease	Linear decrease	Quadratic—U-shaped*	—	Linear decrease
Sweden^a^	Linear increase	Linear increase	—	Linear increase	Cubic*	Cubic*	Linear increase*	Cubic*
Switzerland^b^	—	Quadratic—U-shaped	—	Linear increase	Linear increase*	Cubic*	Linear increase*	Cubic*
Ukraine^c^	Quadratic—U-shaped	Cubic	Cubic	—	—	—	—	—
USA^b^	Linear decrease	Cubic	—	—	—	—	—	—
Wales^a^	Cubic	Quadratic—U-shaped	—	Linear increase	Quadratic—U-shaped*	Quadratic—U-shaped	Quadratic—U-shaped*	Cubic*
*Total number of countries with*								
Linear decrease	4	1	0	2	1	0	0	1
Linear increase	6	14	7	20	8	4	19	9
Quadratic—inverted U-shaped	0	0	5	2	0	0	2	0
Quadratic—U-shaped	7	13	1	1	15	18	2	11
Cubic	3	9	2	4	10	16	2	16
None (—)	21	4	26	12	4	0	13	1

* = Different trend pattern for 1994–2018 and 1994–2022;

^a^ Data since 1994.

^b^ Data since 1998.

^c^ Data since 2002.

^d^ Data since 2006.

^e^ Data since 2010.

The first published incorrect version of the article has been updated.

