# Supplementary material for: Three Decades of Adolescent Health: Unveiling Global Trends Across 41 Countries in Psychological and Somatic Complaints (1994–2022)
Source: Int J Public Health. 2024 Dec 2;69:1607774. doi: 10.3389/ijph.2024.1607774 (PMC11646713; doi:10.3389/ijph.2024.1607774)
Supplement: Supplementary file 1 [file DataSheet1.docx]

**Supplementary Materials**

Supplementary Figure S1: Comparison cut-offs 1, 2, 3 and 4 or more complaints more than once a week

|  |  |
| --- | --- |
|  |  |

Notes. Solid lines represent prevalence rates for psychological complaints, while dotted lines indicate prevalence rates for somatic complaints. Blue lines represent boys, red lines represent girls.

Supplementary Table S1: Sample size (N) of the 15 year olds country/region, year and gender

|  | Sample size 15 year-olds (N) | | | | | | | | | | | | | | | |
| --- | --- | --- | --- | --- | --- | --- | --- | --- | --- | --- | --- | --- | --- | --- | --- | --- |
|  | 1994 | | 1998 | | 2002 | | 2006 | | 2010 | | 2014 | | 2018 | | 2022 | |
|  | *Boy* | *Girl* | *Boy* | *Girl* | *Boy* | *Girl* | *Boy* | *Girl* | *Boy* | *Girl* | *Boy* | *Girl* | *Boy* | *Girl* | *Boy* | *Girl* |
| Armenia | - | - | - | - | - | - | - | - | 398 | 517 | 434 | 610 | 758 | 826 | 515 | 638 |
| Austria | 1128 | 687 | 610 | 766 | 643 | 634 | 693 | 801 | 885 | 935 | 545 | 719 | 616 | 731 | 1050 | 1426 |
| Belgium - Flanders | 576 | 773 | 797 | 762 | 1027 | 1003 | 831 | 785 | 680 | 546 | 1021 | 696 | 711 | 749 | 1763 | 1750 |
| Belgium - Wallonia | 712 | 964 | 280 | 360 | 631 | 750 | 731 | 683 | 669 | 672 | 924 | 1008 | 822 | 843 | 835 | 979 |
| Bulgaria | - | - | - | - | - | - | 804 | 884 | - | - | 910 | 740 | 685 | 832 | 686 | 570 |
| Canada | 1059 | 1160 | 1134 | 1269 | 528 | 679 | 195 | 1194 | 2685 | 2756 | 2506 | 2467 | 2094 | 2198 | 1672 | 1731 |
| Croatia | - | - | - | - | 619 | 816 | 773 | 857 | 1197 | 1227 | 1031 | 915 | 1052 | 1060 | 769 | 897 |
| Czechia | 606 | 595 | 607 | 622 | 806 | 854 | 842 | 823 | 747 | 775 | 852 | 908 | 1934 | 1880 | 2162 | 2101 |
| Denmark | 667 | 647 | 752 | 794 | 656 | 713 | 762 | 790 | 577 | 649 | 584 | 679 | 392 | 374 | 740 | 788 |
| England | - | - | 899 | 973 | 801 | 963 | 709 | 742 | 494 | 624 | 816 | 792 | 432 | 431 | 571 | 605 |
| Estonia | 549 | 630 | 252 | 335 | 619 | 648 | 801 | 786 | 661 | 737 | 638 | 631 | 759 | 783 | 812 | 740 |
| Finland | 576 | 618 | 770 | 775 | 867 | 874 | 790 | 895 | 1008 | 1102 | 956 | 1009 | 531 | 545 | 456 | 514 |
| France | 620 | 640 | 592 | 653 | 1301 | 1313 | 1139 | 1083 | 904 | 1002 | 865 | 875 | 1114 | 1212 | 964 | 886 |
| Germany | 515 | 535 | 799 | 800 | 842 | 899 | 1271 | 1281 | 736 | 904 | 1061 | 1043 | 659 | 858 | 948 | 1094 |
| Greece | - | - | 603 | 719 | 643 | 681 | 650 | 766 | 842 | 806 | 638 | 682 | 659 | 651 | 1077 | 1310 |
| Greenland | 178 | 197 | 311 | 288 | 100 | 138 | 207 | 210 | 189 | 208 | 157 | 163 | 168 | 146 | 194 | 242 |
| Hungary | 825 | 934 | 374 | 444 | 498 | 812 | 550 | 637 | 799 | 934 | 535 | 565 | 506 | 639 | 730 | 948 |
| Iceland | - | - | - | - | - | - | 947 | 936 | 1873 | 1807 | 1659 | 1657 | 1074 | 1104 | 1420 | 1353 |
| Ireland | - | - | 745 | 712 | 345 | 574 | 914 | 771 | 962 | 733 | 576 | 944 | 529 | 554 | 478 | 603 |
| Israel | 667 | 685 | 649 | 736 | 697 | 850 | 758 | 1239 | 681 | 671 | 857 | 1007 | 1149 | 1558 | 1671 | 1981 |
| Italy | - | - | - | - | 541 | 679 | 678 | 657 | 764 | 782 | 640 | 622 | 581 | 720 | 845 | 703 |
| Latvia | 472 | 791 | 508 | 757 | 481 | 631 | 628 | 702 | 666 | 709 | 784 | 942 | 660 | 682 | 1020 | 1024 |
| Lithuania | 788 | 971 | 636 | 799 | 981 | 923 | 940 | 921 | 945 | 847 | 904 | 794 | 572 | 610 | 935 | 883 |
| Luxembourg | - | - | - | - | - | - | 776 | 731 | 702 | 680 | 499 | 580 | 653 | 683 | 728 | 725 |
| North-Macedonia | - | - | - | - | 672 | 727 | 952 | 944 | 814 | 722 | 742 | 715 | 736 | 760 | 802 | 806 |
| Malta | - | - | - | - | 310 | 349 | 184 | 170 | - | - | 318 | 327 | 345 | 383 | 409 | 443 |
| Netherlands | - | - | - | - | 637 | 636 | 672 | 691 | 730 | 727 | 658 | 699 | 720 | 803 | 650 | 652 |
| Norway | 837 | 800 | 848 | 822 | 799 | 823 | 818 | 716 | 711 | 628 | 467 | 503 | 327 | 350 | 438 | 412 |
| Poland | 809 | 731 | 891 | 745 | 1022 | 1105 | 1092 | 1195 | 685 | 725 | 700 | 784 | 852 | 929 | 998 | 1206 |
| Portugal | - | - | 486 | 759 | 378 | 422 | 613 | 770 | 680 | 873 | 630 | 730 | 664 | 751 | 778 | 984 |
| Romania | - | - | - | - | - | - | 606 | 999 | 1046 | 956 | 633 | 809 | 752 | 817 | 1373 | 1560 |
| Russia | 631 | 723 | 612 | 710 | 1138 | 1436 | 1238 | 1516 | 919 | 928 | 659 | 786 | 852 | 966 | - | - |
| Scotland | 641 | 732 | 810 | 917 | 578 | 571 | 1108 | 1090 | 1232 | 1335 | 951 | 918 | 684 | 735 | 597 | 541 |
| Slovakia | 478 | 456 | 503 | 340 | - | - | 591 | 661 | 961 | 953 | 971 | 864 | 703 | 590 | 895 | 723 |
| Slovenia | - | - | - | - | 543 | 509 | 780 | 781 | 914 | 901 | 744 | 871 | 898 | 815 | 997 | 1174 |
| Spain | 688 | 799 | - | - | 821 | 935 | 1519 | 1546 | 962 | 1041 | 1761 | 1998 | 753 | 792 | 699 | 782 |
| Sweden | 591 | 560 | 610 | 541 | 609 | 609 | 752 | 774 | 1059 | 1031 | 1358 | 1408 | 771 | 825 | 645 | 604 |
| Switzerland | - | - | 918 | 914 | 770 | 731 | 733 | 767 | 1138 | 1108 | 1100 | 1112 | 1208 | 1186 | 1066 | 1129 |
| Ukraine | - | - | - | - | 730 | 871 | 835 | 994 | 881 | 1016 | 792 | 902 | 1042 | 983 | - | - |
| USA | - | - | 813 | 995 | 754 | 871 | 649 | 635 | 968 | 924 | - | - | - | - | - | - |
| Wales | 599 | 667 | 723 | 704 | 603 | 561 | 675 | 675 | 855 | 782 | 729 | 703 | 2167 | 2137 | 5171 | 5048 |
| Total | 15212 | 16295 | 18532 | 20011 | 23990 | 26590 | 31206 | 34098 | 34619 | 35273 | 33605 | 35177 | 32584 | 34491 | 38559 | 40555 |

Supplementary Table S2: Prevalence of two or more psychological and somatic complaints in boys and girls by country and year

|  | % 2 or more psychological complaints more than weekly | | | | | | | | | | | | | | | | % 2 or more somatic complaints more than weekly | | | | | | | | | | | | | | | |
| --- | --- | --- | --- | --- | --- | --- | --- | --- | --- | --- | --- | --- | --- | --- | --- | --- | --- | --- | --- | --- | --- | --- | --- | --- | --- | --- | --- | --- | --- | --- | --- | --- |
|  | *Boys* | | | | | | | | *Girls* | | | | | | | | *Boys* | | | | | | | | *Girls* | | | | | | | |
|  | ‘94 | ‘98 | ‘02 | ‘06 | ‘10 | ‘14 | ‘18 | ‘22 | ‘94 | ‘98 | ‘02 | ‘06 | ‘10 | ‘14 | ‘18 | ‘22 | ‘94 | ‘98 | ‘02 | ‘06 | ‘10 | ‘14 | ‘18 | ‘22 | ‘94 | ‘98 | ‘02 | ‘06 | ‘10 | ‘14 | ‘18 | ‘22 |
| Armenia | - | - | - | - | 28.8 | 26.2 | 28.1 | 30.5 | - | - | - | - | 44.9 | 36.2 | 44.1 | 55.4 | - | - | - | - | 10.5 | 11.9 | 8.9 | 11.4 | - | - | - | - | 18.8 | 13.7 | 11.9 | 20.2 |
| Austria | 9.0 | 4.9 | 10.3 | 7.5 | 9.8 | 11.7 | 14.8 | 23.2 | 13.7 | 12.8 | 13.1 | 15.3 | 18.2 | 20.5 | 33.4 | 48.0 | 5.7 | 2.5 | 5.4 | 4.5 | 7.9 | 5.5 | 7.0 | 9.3 | 12.0 | 11.6 | 12.0 | 13.3 | 15.9 | 14.5 | 19.4 | 30.1 |
| Belgium - Flanders | 10.9 | 13.8 | 16.8 | 18.3 | 13.7 | 13.3 | 15.6 | 22.8 | 16.8 | 19.8 | 21.7 | 18.3 | 19.8 | 29.8 | 28.6 | 49.9 | 5.1 | 4.1 | 7.4 | 9.0 | 8.3 | 10.3 | 5.0 | 10.5 | 9.4 | 10.5 | 14.4 | 13.2 | 19.7 | 23.9 | 15.8 | 32.0 |
| Belgium - Wallonia | 27.6 | 28.2 | 20.8 | 21.5 | 18.3 | 26.0 | 28.7 | 34.3 | 34.2 | 45.5 | 32.6 | 36.0 | 36.0 | 40.1 | 47.7 | 60.5 | 6.4 | 9.9 | 12.3 | 10.4 | 12.4 | 11.9 | 9.5 | 13.6 | 17.9 | 23.2 | 22.2 | 21.2 | 27.1 | 24.9 | 25.4 | 34.5 |
| Bulgaria | - | - | - | 23.8 | - | 28.9 | 42.3 | 30.2 | - | - | - | 44.3 | - | 50.1 | 53.1 | 59.5 | - | - | - | 11.0 | - | 11.0 | 18.7 | 18.7 | - | - | - | 18.4 | - | 23.1 | 21.3 | 34.4 |
| Canada | 22.4 | 14.5 | 20.6 | 17.5 | 17.5 | 19.8 | 20.0 | 28.5 | 27.1 | 24.7 | 25.6 | 29.3 | 31.5 | 39.8 | 43.0 | 58.9 | 11.2 | 7.8 | 11.8 | 9.5 | 10.7 | 10.2 | 8.6 | 13.4 | 22.0 | 19.7 | 18.4 | 21.2 | 23.4 | 26.2 | 26.2 | 38.9 |
| Croatia | - | - | 20.1 | 18.8 | 17.9 | 15.7 | 17.6 | 20.7 | - | - | 37.3 | 36.2 | 28.4 | 36.0 | 37.7 | 50.1 | - | - | 6.2 | 5.7 | 5.6 | 6.0 | 5.4 | 6.1 | - | - | 17.0 | 15.3 | 14.6 | 20.1 | 15.8 | 24.8 |
| Czechia | - | 18.1 | 15.4 | 19.7 | 26.9 | 21.9 | 21.9 | 28.3 | - | 28.7 | 25.9 | 31.4 | 36.8 | 38.5 | 39.6 | 55.7 | - | 6.6 | 6.8 | 5.4 | 9.8 | 5.3 | 4.5 | 7.2 | - | 11.5 | 11.7 | 14.3 | 19.2 | 16.8 | 13.8 | 22.3 |
| Denmark | 6.5 | 8.6 | 9.4 | 9.8 | 11.4 | 13.0 | 14.1 | 17.3 | 14.2 | 21.7 | 17.1 | 21.4 | 16.9 | 28.7 | 28.7 | 42.6 | 2.6 | 4.4 | 4.0 | 3.5 | 6.7 | 5.8 | 7.3 | 9.9 | 6.5 | 9.2 | 8.6 | 14.2 | 9.6 | 14.6 | 13.4 | 26.9 |
| England | - | 17.4 | 20.4 | 19.3 | 14.0 | 20.8 | 31.6 | 36.2 | - | 24.6 | 28.9 | 31.5 | 32.8 | 37.8 | 48.4 | 68.4 | - | 7.6 | 10.9 | 6.6 | 10.6 | 7.8 | 8.4 | 17.0 | - | 12.4 | 20.0 | 18.0 | 21.0 | 23.3 | 24.8 | 40.7 |
| Estonia | 18.0 | 18.3 | 18.4 | 21.1 | 16.3 | 18.4 | 25.4 | 33.3 | 29.6 | 35.8 | 38.6 | 30.7 | 28.6 | 36.2 | 46.3 | 63.3 | 5.9 | 9.6 | 8.3 | 7.6 | 11.0 | 6.7 | 11.5 | 17.5 | 11.7 | 19.0 | 24.4 | 16.4 | 17.3 | 18.3 | 25.7 | 39.7 |
| Finland | 14.0 | 14.5 | 12.4 | 16.1 | 12.4 | - | 21.3 | 18.7 | 21.8 | 20.0 | 22.8 | 25.4 | 26.5 | - | 39.0 | 51.6 | 4.6 | 5.7 | 6.1 | 7.8 | 6.4 | - | 10.3 | 7.2 | 12.5 | 13.6 | 17.7 | 17.1 | 16.2 | - | 19.8 | 31.1 |
| France | 23.5 | 24.1 | 18.1 | 19.5 | 19.6 | 24.8 | 26.8 | 28.9 | 37.6 | 42.6 | 32.1 | 37.5 | 34.2 | 43.0 | 46.0 | 59.7 | 6.1 | 6.2 | 5.3 | 8.8 | 9.4 | 11.9 | 8.6 | 12.7 | 11.0 | 21.5 | 14.3 | 22.5 | 19.9 | 26.2 | 18.9 | 33.2 |
| Germany | 9.4 | 10.3 | 7.3 | 10.5 | 8.6 | 10.7 | 13.1 | 22.9 | 17.3 | 17.1 | 13.7 | 18.2 | 16.1 | 25.7 | 26.3 | 56.0 | 4.6 | 4.2 | 4.8 | 6.2 | 5.3 | 6.2 | 6.2 | 9.6 | 14.1 | 12.0 | 12.3 | 14.8 | 15.1 | 20.9 | 18.7 | 32.0 |
| Greece | - | 37.3 | 37.8 | 30.7 | 33.0 | 23.6 | 31.7 | 43.0 | - | 51.7 | 56.5 | 50.6 | 49.1 | 47.3 | 54.9 | 73.6 | - | 7.4 | 8.5 | 5.8 | 8.3 | 7.5 | 9.9 | 13.8 | - | 18.3 | 19.9 | 17.9 | 17.3 | 19.4 | 19.1 | 39.9 |
| Greenland | 13.0 | 12.4 | 15.4 | 12.1 | 16.6 | 19.0 | 21.7 | 22.1 | 17.4 | 24.6 | 24.4 | 32.8 | 33.5 | 31.6 | 42.7 | 47.8 | 5.2 | 7.1 | 5.5 | 8.1 | 10.4 | 13.1 | 20.2 | 11.7 | 6.7 | 13.3 | 12.2 | 15.6 | 18.6 | 26.6 | 25.0 | 24.6 |
| Hungary | 24.7 | 20.6 | 22.0 | 25.2 | 21.7 | 21.2 | 27.9 | 36.2 | 36.6 | 35.5 | 34.6 | 31.6 | 28.1 | 35.8 | 44.4 | 63.2 | 5.2 | 8.1 | 6.1 | 9.0 | 11.3 | 11.3 | 11.2 | 14.8 | 13.2 | 19.1 | 22.0 | 21.5 | 21.0 | 27.2 | 28.4 | 42.1 |
| Iceland | - | - | - | 22.3 | 17.9 | 18.2 | 20.5 | 23.5 | - | - | - | 33.7 | 30.2 | 37.1 | 37.5 | 55.0 | - | - | - | 13.7 | 11.5 | 11.1 | 12.3 | 13.6 | - | - | - | 28.5 | 21.9 | 27.2 | 25.2 | 40.5 |
| Ireland | - | 21.2 | 17.4 | 17.1 | 20.0 | 19.4 | 23.3 | 31.8 | - | 24.2 | 28.4 | 28.2 | 30.6 | 40.4 | 40.0 | 61.0 | - | 7.6 | 6.2 | 8.8 | 9.9 | 10.0 | 9.4 | 17.3 | - | 9.4 | 13.3 | 16.1 | 22.2 | 24.7 | 22.7 | 39.0 |
| Israel | 41.5 | 37.7 | 39.2 | 41.4 | 36.1 | 33.0 | 35.3 | 39.8 | 43.1 | 46.0 | 47.0 | 49.6 | 44.5 | 41.4 | 47.1 | 62.5 | 13.7 | 20.4 | 16.7 | 21.6 | 18.5 | 17.8 | 21.1 | 15.4 | 27.4 | 28.9 | 28.7 | 33.1 | 32.4 | 30.7 | 30.2 | 42.4 |
| Italy | - | - | 29.1 | 31.6 | 28.1 | 31.8 | 35.3 | 40.6 | - | - | 49.5 | 50.0 | 51.7 | 57.2 | 63.4 | 80.2 | - | - | 9.0 | 11.4 | 10.3 | 10.6 | 11.5 | 13.5 | - | - | 24.7 | 27.1 | 32.7 | 31.1 | 32.9 | 46.1 |
| Latvia | 14.2 | 20.6 | 14.9 | 18.5 | 15.4 | 20.8 | 23.3 | 30.0 | 31.0 | 34.5 | 33.7 | 40.7 | 33.0 | 41.6 | 49.0 | 65.4 | 1.7 | 4.2 | 5.1 | 6.6 | 8.6 | 9.2 | 9.4 | 10.9 | 14.0 | 14.4 | 18.0 | 17.0 | 17.7 | 24.6 | 25.4 | 38.2 |
| Lithuania | 20.1 | 19.1 | 19.3 | 23.9 | 20.1 | 16.8 | 22.0 | 30.0 | 36.9 | 41.2 | 45.7 | 44.0 | 35.3 | 39.2 | 40.5 | 63.7 | 5.5 | 9.1 | 9.5 | 13.0 | 11.3 | 9.5 | 8.9 | 11.0 | 16.8 | 24.6 | 23.8 | 24.0 | 20.2 | 22.6 | 22.1 | 32.7 |
| Luxembourg | - | - | - | 19.2 | 20.4 | 24.6 | 23.4 | 27.3 | - | - | - | 38.0 | 33.0 | 42.7 | 36.7 | 56.0 | - | - | - | 8.4 | 12.0 | 12.4 | 8.2 | 11.3 | - | - | - | 23.5 | 22.0 | 27.1 | 21.4 | 31.4 |
| North-Macedonia | - | - | 22.5 | 20.8 | 18.1 | 25.2 | - | 29.9 | - | - | 41.9 | 38.3 | 33.8 | 45.6 | - | 56.1 | - | - | 5.9 | 6.7 | 6.2 | 8.2 | - | 12.3 | - | - | 16.8 | 12.4 | 15.0 | 15.3 | - | 25.1 |
| Malta | - | - | 21.9 | 35.4 |  | 31.9 | 29.8 | 34.8 | - | - | 32.1 | 46.6 |  | 45.3 | 49.3 | 62.9 | - | - | 6.6 | 18.0 | - | 19.7 | 17.9 | 19.7 | - | - | 13.8 | 23.3 |  | 32.8 | 31.5 | 38.5 |
| Netherlands | - | - | 9.7 | 9.5 | 7.6 | 12.9 | 13.2 | 21.1 | - | - | 18.4 | 21.9 | 17.5 | 28.8 | 27.1 | 43.9 | - | - | 6.0 | 5.8 | 4.2 | 6.8 | 7.4 | 9.9 | - | - | 13.4 | 15.5 | 13.8 | 25.2 | 17.8 | 28.4 |
| Norway | 9.4 | 11.2 | 12.4 | 13.6 | 12.3 | 11.6 | 16.6 | 24.9 | 15.3 | 19.2 | 24.0 | 24.6 | 30.9 | 29.5 | 32.9 | 47.6 | 6.1 | 5.7 | 4.6 | 6.4 | 8.0 | 6.9 | 8.3 | 8.9 | 12.6 | 13.5 | 16.1 | 13.2 | 18.6 | 17.7 | 15.6 | 24.1 |
| Poland | 14.8 | 24.1 | 25.1 | 22.9 | 24.6 | 26.7 | 34.8 | 38.4 | 30.0 | 39.6 | 38.2 | 35.7 | 37.6 | 43.8 | 49.6 | 64.5 | 3.7 | 7.0 | 7.8 | 7.5 | 9.5 | 16.2 | 6.4 | 13.1 | 10.3 | 15.8 | 15.5 | 17.3 | 23.3 | 27.2 | 19.8 | 39.0 |
| Portugal | - | 17.3 | 14.4 | 9.4 | 12.5 | 11.5 | 19.5 | 25.3 | - | 30.4 | 33.8 | 21.8 | 23.2 | 31.9 | 43.0 | 56.8 | - | 6.9 | 5.0 | 4.2 | 5.5 | 6.7 | 6.3 | 8.2 | - | 17.6 | 15.2 | 14.9 | 15.3 | 16.5 | 20.3 | 28.8 |
| Romania | - | - | - | 28.4 | 24.9 | 20.5 | 26.7 | 36.5 | - | - | - | 51.8 | 44.0 | 41.6 | 48.5 | 57.5 | - | - | - | 9.9 | 12.5 | 11.4 | 10.3 | 12.7 | - | - | - | 25.1 | 24.7 | 26.3 | 20.3 | 32.9 |
| Russia | 17.3 | 22.9 | 17.6 | 19.9 | 18.1 | 20.6 | 19.5 | - | 35.5 | 36.6 | 29.0 | 31.6 | 32.2 | 37.2 | 34.3 | - | 3.8 | 8.8 | 7.7 | 13.5 | 11.9 | 12.9 | 11.5 | - | 11.8 | 18.7 | 15.4 | 18.6 | 23.3 | 19.6 | 19.1 | - |
| Scotland | 16.3 | 13.6 | 16.6 | 13.4 | 18.9 | 21.1 | 29.2 | 32.9 | 25.7 | 23.6 | 26.8 | 25.8 | 29.2 | 44.0 | 47.5 | 59.6 | 6.5 | 8.3 | 5.7 | 6.5 | 9.0 | 8.8 | 10.5 | 11.4 | 15.8 | 21.0 | 15.5 | 13.2 | 20.0 | 25.2 | 26.2 | 39.3 |
| Slovakia | 16.7 | 19.8 | - | 30.4 | 23.5 | 22.6 | 26.4 | 32.4 | 30.5 | 34.9 | - | 38.2 | 31.0 | 36.6 | 40.8 | 60.3 | 3.0 | 5.7 |  | 8.8 | 9.4 | 12.7 | 9.4 | 10.7 | 12.3 | 18.6 | - | 15.1 | 19.7 | 22.8 | 21.0 | 35.0 |
| Slovenia | - | - | 13.2 | 12.0 | 8.7 | 16.0 | 18.3 | 21.5 | - | - | 28.3 | 18.8 | 16.4 | 39.9 | 45.5 | 50.9 | - | - | 4.1 | 7.0 | 4.1 | 6.7 | 5.3 | 8.0 | - | - | 11.7 | 8.1 | 10.1 | 16.8 | 17.5 | 22.0 |
| Spain | - | - | 21.8 | 18.1 | 18.3 | 15.6 | 13.6 | 16.7 | - | - | 40.5 | 32.7 | 32.4 | 33.1 | 31.2 | 46.1 | - | - | 11.0 | 8.5 | 8.7 | 8.9 | 4.9 | 7.7 | - | - | 25.7 | 21.1 | 18.3 | 20.9 | 16.2 | 22.3 |
| Sweden | 14.8 | 20.3 | 19.3 | 20.9 | 16.0 | 23.4 | 26.0 | 35.5 | 27.3 | 29.3 | 39.1 | 41.6 | 34.4 | 45.6 | 50.9 | 66.2 | 6.1 | 10.5 | 7.6 | 11.1 | 8.7 | 10.2 | 9.9 | 15.4 | 14.7 | 21.5 | 19.6 | 24.5 | 22.7 | 25.1 | 27.0 | 41.0 |
| Switzerland | - | 11.0 | 9.8 | 14.8 | 13.3 | 13.6 | 14.7 | 20.9 | - | 28.5 | 20.9 | 27.7 | 23.9 | 29.5 | 32.0 | 52.4 | - | 4.6 | 4.8 | 6.7 | 5.9 | 6.9 | 6.0 | 10.8 | - | 13.3 | 12.9 | 16.0 | 17.3 | 16.8 | 18.6 | 36.1 |
| Ukraine | - | - | 23.7 | 23.2 | 17.4 | 16.6 | 24.5 | - | - | - | 47.8 | 42.0 | 45.5 | 31.1 | 47.2 | - | - | - | 5.6 | 12.1 | 7.2 | 7.4 | 7.8 | - | - | - | 19.4 | 20.3 | 21.4 | 15.4 | 22.1 | - |
| USA | - | 27.3 | 20.5 | 22.6 | 15.8 | - | - | - | - | 36.5 | 29.9 | 39.1 | 29.8 | - | - | - | - | 15.6 | 13.2 | 10.4 | 10.9 | - | - | - | - | 26.6 | 26.0 | 31.1 | 23.3 | - | - | - |
| Wales | 14.1 | 20.2 | 17.1 | 14.0 | 14.6 | 18.2 | 26.4 | 33.5 | 25.3 | 26.1 | 28.7 | 28.1 | 27.7 | 36.8 | 48.5 | 58.8 | 7.7 | 11.0 | 7.3 | 5.9 | 7.5 | 8.6 | 9.6 | 13.4 | 13.7 | 19.6 | 18.3 | 16.9 | 18.7 | 24.2 | 25.1 | 38.4 |

Notes. ‘ –‘ no data available

Supplementary Table S3: Sensitivity testing cut-off 1, 2 or 3 or more complaints more than once a week on the pooled sample by sex and health complaint dimension

|  | Boys Psychological complaints | | | Girls Psychological complaints | | | Boys Somatic complaints | | | Girls Somatic complaints | | |
| --- | --- | --- | --- | --- | --- | --- | --- | --- | --- | --- | --- | --- |
|  | *Linear trend* | *Quadratic trend* | *Cubic trend* | *Linear trend* | *Quadratic trend* | *Cubic trend* | *Linear trend* | *Quadratic trend* | *Cubic trend* | *Linear trend* | *Quadratic trend* | *Cubic trend* |
| One or more complaints | 0.004(0.000)*** | 13.487(0.487)*** | 6.543(0.487)*** | 0.008(0.000)*** | 20.203(0.488)*** | 9.897(0.487)*** | 0.003(0.00)*** | 4.126(0.435)*** | 4.0153(0.435)*** | 0.007(0.00)*** | 11.134(0.494)*** | 8.839(0.493)*** |
| Two or more complaints | 0.004(0.000)*** | 9.924(0.407)*** | 5.667(0.407)*** | 0.009(0.000)*** | 20.441(0.477)*** | 11.839(0.476)*** | 0.002(0.000)*** | 0.740(0.288)** | 1.954(0.288)*** | 0.006(0.000)*** | 9.793(0.410)*** | 8.549(0.410)*** |
| Three or more complaints | 0.003(0.000)*** | 6.060(0.304)*** | 3.611(0.304)*** | 0.009(0.000)*** | 16.603(0.413)*** | 10.541(0.413)*** | 0.001(0.000)*** | -0.388(0.186)* | 0.606(0.186)*** | 0.004(0.000)*** | 6.744(0.292)*** | 5.127(0.291)*** |

Notes. Estimates(Standard error); P<0.001 = ***; P<0.01=**; P<0.05=*

Supplementary Table S4: Sensitivity testing time trend pattern for psychological complaints by country and sex

|  | Boys psychological complaints | | | | Girls psychological complaints | | | | Trend pattern | | |  |
| --- | --- | --- | --- | --- | --- | --- | --- | --- | --- | --- | --- | --- |
|  | | **Linear trend** | **Quadratic trend** | **Cubic trend** | | **Linear trend** | **Quadratic trend** | **Cubic trend** | | **Boys** | **Girls** | |
| Armenia | | 0.000(0.004) | 0.358(0.448) | - | | 0.001(0.004) | 1.601(0.492)** | - | | / | / | |
| Austria | | 0.002(0.001)*** | 0.916(0.294)** | -0.077(0.294) | | 0.007(0.001)*** | 2.389(0.380)*** | 0.729(0.380) | | LI | QU | |
| Belgium - Flanders | | 0.001(0.001) | -0.985(0.355)** | 0.963(0.355)** | | 0.005(0.001)*** | 0.724(0.413) | 0.394(0.413) | | / | LI | |
| Belgium - Wallonia | | 0.000(0.001) | 2.228(0.428)*** | 0.284(0.428) | | 0.004(0.001)*** | 1.734(0.485)*** | 1.213(0.485)* | | QU | QU | |
| Bulgaria | | 0.014(0.002)*** | 1.662(0.457)*** | - | | 0.007(0.002)*** | 0.020.499) | - | | QU | LI | |
| Canada | | 0.000(0.000) | 1.239(0.391)*** | -0.933(0.390)* | | 0.008(0.001)*** | 2.579(0.468)*** | -0.831(0.468) | | / | QU | |
| Croatia | | -0.002(0.001) | 0.399(0.383) | 0.345(0.383) | | 0.000(0.001) | 1.959(0.475)*** | 0.046(0.475) | | / | QU | |
| Czechia | | 0.003(0.001)*** | -0.897(0.406)* | -1.081(0.406)** | | 0.007(0.001)*** | -0.062(0.474) | -1.175(0.474)* | | LI | LI | |
| Denmark | | 0.003(0.001)*** | 0.034(0.300) | 0.146(0.300) | | 0.005(0.001)*** | 0.708(0.405) | 0.902(0.404)* | | LI | LI | |
| England | | 0.004(0.001)*** | 1.463(0.399)*** | 1.649(0.398)*** | | 0.010(0.001)*** | 0.921(0.463)* | 1.042(0.463)* | | C | LI | |
| Estonia | | 0.002(0.001)** | 0.800(0.398)* | 1.053(0.398)** | | 0.004(0.001)*** | 1.814(0.476)*** | 2.722(0.474)*** | | / | C | |
| Finland | | 0.002(0.001)** | 1.091(0.353)** | 0.612(0.353) | | 0.007(0.001)*** | 1.534(0.432)*** | 0.205(0.432) | | / | QU | |
| France | | 0.002(0.001)** | 2.029(0.413)*** | -0.106(0.413) | | 0.004(0.001)*** | 2.509(0.485)*** | 0.319(0.486) | | QU | QU | |
| Germany | | 0.001(0.001)* | 0.578(0.300) | 0.183(0.300) | | 0.005(0.001)*** | 1.661(0.393)*** | -0.344(0.394) | | / | QU | |
| Greece | | -0.005(0.001)*** | 0.747(0.467) | 1.092(0.466)* | | -0.001(0.001) | 0.866(0.500) | 1.662(0.499)*** | | LI | C | |
| Greenland | | 0.004(0.001)** | 0.433(0.358) | -0.006(0.358) | | 0.009(0.002)*** | -0.162(0.449) | 0.498(0.449) | | / | LI | |
| Hungary | | 0.000(0.001) | 0.869(0.423)* | 0.339(0.423) | | 0.001(0.001) | 2.698(0.475)*** | 1.664(0.475)*** | | / | C | |
| Iceland | | -0.001(0.001) | 1.192(0.394)** | -0.234(0.394) | | 0.006(0.002)*** | 0.710(0.474 | -1.503(0.474)** | | / | LI | |
| Ireland | | 0.001(0.001) | 1.046(0.397)** | -0.314(0.397) | | 0.009(0.001)*** | 0.535(0.464) | -0.442(0.464) | | / | LI | |
| Israel | | -0.003(0.001)*** | -0.121(0.484) | 0.265(0.484) | | 0.000(0.001) | -0.624(0.498) | 1.449(0.498)** | | LD | / | |
| Italy | | 0.003(0.002)* | 0.666(0.462) | 0.475(0.462) | | 0.009(0.001)*** | 1.083(0.496)* | -0.040(0.496) | | / | LI | |
| Latvia | | 0.003(0.001)*** | 0.648(0.388) | 0.782(0.388)* | | 0.006(0.001)*** | 1.026(0.482)* | 1.279(0.482)** | | LI | LI | |
| Lithuania | | 0.000(0.001) | -0.263(0.401) | 0.364(0.401) | | 0.000(0.001) | -1.215(0.491)* | 1.896(0.490)*** | | / | C | |
| Luxembourg | | 0.004(0.002)* | -0.235(0.411) | -0.442(0.411) | | 0.001(0.002) | -0.021(0.484) | -1.695(0.483)*** | | / | C | |
| North-Macedonia | | 0.001(0.002) | 1.156(0.410)** | 0.701(0.410) | | 0.002(0.002) | 2.0100.488)*** | 1.088(*0.488)* | | / | QU | |
| Malta | | 0.004(0.002) | -1.134(0.453)* | 0.725(0.453) | | 0.009(0.002)*** | -0.636(0.492) | 0.959(0.491) | | / | LI | |
| Netherlands | | 0.003(0.001)** | 0.599(0.307) | -0.285(0.307) | | 0.006(0.001)*** | 0.398(0.419) | -0.463(0.419) | | / | LI | |
| Norway | | 0.002(0.001)** | -0.159(0.325) | 0.561(0.325) | | 0.007(0.001)*** | -0.608(0.423) | 0.021(0.423) | | / | LI | |
| Poland | | 0.006(0.001)*** | 0.242(0.429) | 2.108(0.428)*** | | 0.006(0.001)*** | 1.022(0.486)* | 1.820(0.486)*** | | C | C | |
| Portugal | | 0.001(0.001) | 1.890(0.346)*** | 0.281(0.346) | | 0.005(0.001)*** | 3.803(0.454)*** | 1.030(0.454)* | | QU | QU | |
| Romania | | -0.002(0.002) | 1.196(0.433)** | 0.706(0.433) | | -0.003(0.002) | 2.161(0.498)*** | 0.250(0.498) | | / | QU | |
| Russia | | 0.000(0.001) | -0.014(0.394) | 0.289(0.394) | | 0.000(0.001) | 1.455(0.470)** | -0.862(0.470) | | / | / | |
| Scotland | | 0.005(0.001)*** | 2.112(0.383)*** | 0.216(0.383) | | 0.010(0.001)*** | 3.276(0.457)*** | -0.066(0.457) | | QU | QU | |
| Slovakia | | 0.003(0.001)*** | -1.012(0.423)* | 1.065(0.423)* | | 0.003(0.001)** | 0.495(0.477) | 1.357(0.477)** | | LI | / | |
| Slovenia | | 0.004(0.001)*** | 1.345(0.341)*** | -0.261(0.341) | | 0.016(0.001)*** | 3.930(0.445)*** | -2.314(0.443)*** | | QU | C | |
| Spain | | -0.004(0.001)*** | 0.067(0.378) | -0.363(0.378) | | -0.004(0.001)*** | 1.094(0.472)* | -1.199(0.472)* | | LD | LD | |
| Sweden | | 0.003(0.001)*** | 0.664(0.402) | 1.208(0.402)** | | 0.009(0.001)*** | 0.464(0.485) | 1.442(0.485)** | | LI | LI | |
| Switzerland | | 0.002(0.001)** | -0.173(0.336) | -0.032(0.336) | | 0.003(0.001)*** | 1.632(0.445)*** | -0.581(0.446) | | / | QU | |
| Ukraine | | -0.001(0.001) | 1.726(0.408)*** | 1.235(0.408)** | | -0.003(0.001)* | 2.033(0.494)*** | 1.937(0.493)*** | | QU | C | |
| USA | | -0.008(0.002)*** | 0.019(0.407) | -1.045(0.407)** | | -0.003(0.002) | -0.150(0.472) | -2.103(0.471)*** | | LD | C | |
| Wales | | 0.004(0.001)*** | 2.119(0.396)*** | 1.848(0.396)*** | | 0.010(0.001)*** | 3.240(0.469)*** | 1.490(0.469)** | | C | QU | |

Notes. Estimates(Standard error); P<0.001 = ***; P<0.01=**; P<0.05=*; LI = linear increase; LD= linear decrease; QU = quadratic U-shaped; QIU = quadratic inverted U-shaped; C = cubic

Supplementary Table S5: Sensitivity testing time trend pattern for somatic complaints by country and sex

|  | Boys somatic complaints | | | | Girls somatic complaints | | | | Trend pattern | | |  |
| --- | --- | --- | --- | --- | --- | --- | --- | --- | --- | --- | --- | --- |
|  | | **Linear trend** | **Quadratic trend** | **Cubic trend** | | **Linear trend** | **Quadratic trend** | **Cubic trend** | | **Boys** | **Girls** | |
| Armenia | | -0.003(0.003) | -0.345(0.301) | - | | -0.008(0.003)*** | 0.300(0.349) | - | | / | LD | |
| Austria | | 0.001(0.000)* | 0.217(0.230) | -0.552(0.230)* | | 0.003(0.001)*** | 0.568(0.348) | 0.030(0.348) | | / | LI | |
| Belgium - Flanders | | 0.001(0.000)** | -0.975(0.259)*** | -0.833(0.259)** | | 0.004(0.001)*** | -0.814(0.354)* | -1.287(0.354)*** | | QIU | C | |
| Belgium - Wallonia | | 0.001(0.001)* | -1.046(0.305)*** | 0.031(0.305) | | 0.003(0.001)*** | -0.525(0.420) | -0.002(0.420) | | QIU | LI | |
| Bulgaria | | 0.005(0.001)*** | 1.174(0.337)*** | - | | 0.003(0.002) | -0.627(0.406) | - | | QU | / | |
| Canada | | 0.000(0.000) | -0.345(0.299) | -0.772(0.298)** | | 0.003(0.001)*** | 0.958(0.423)* | -1.111(0.423)** | | / | LI | |
| Croatia | | 0.000(0.001) | 0.017(0.233) | -0.135(0.233) | | 0.001(0.001) | 0.132(0.370) | -1.022(0.370)** | | / | / | |
| Czechia | | -0.001(0.000)** | -0.564(0.237)* | -0.347(0.237) | | 0.001(0.001)* | -1.321(0.351)*** | -0.911(0.351)** | | / | QIU | |
| Denmark | | 0.002(0.000)*** | 0.117(0.210) | 0.086(0.210) | | 0.003(0.001)*** | -0.340(0.309) | 0.057(0.309) | | LI | LI | |
| England | | 0.000(0.001) | -0.194(0.280) | 0.278(0.280) | | 0.006(0.001)*** | -0.519(0.392) | 0.663(0.392) | | / | LI | |
| Estonia | | 0.002(0.001)** | 0.042(0.282) | 0.479(0.282) | | 0.003(0.001)*** | 0.066(0.392) | 2.254(0.391)*** | | / | C | |
| Finland | | 0.002(0.001)*** | 0.169(0.249) | 0.331(0.249) | | 0.003(0.001)*** | -0.238(0.367) | 0.574(0.367) | | LI | LI | |
| France | | 0.002(0.000)*** | -0.240(0.272) | -0.970(0.272)*** | | 0.003(0.001)*** | -1.343(0.393)*** | -0.756(0.393) | | C | QIU | |
| Germany | | 0.001(0.000)* | -0.096(0.228) | -0.094(0.228) | | 0.003(0.001)*** | 0.604(0.362) | -0.851(0.362)* | | / | LI | |
| Greece | | 0.001(0.001) | 0.369(0.270) | 0.156(0.270) | | 0.000(0.001) | 0.207(0.389) | 0.195(0.389) | | / | / | |
| Greenland | | 0.005(0.001)*** | 0.583(0.292)* | 0.254(0.292) | | 0.008(0.001)*** | 0.074(0.365) | 0.053(0.365) | | LI | LI | |
| Hungary | | 0.003(0.001)*** | -0.195(0.282) | -0.287(0.282) | | 0.006(0.001)*** | -0.224(0.407) | 1.008(0.407)* | | LI | LI | |
| Iceland | | -0.001(0.001) | 0.625(0.324) | -0.036(0.324) | | 0.000(0.001) | 0.821(0.435) | -1.705(0.434)*** | | / | C | |
| Ireland | | 0.001(0.001)* | -0.187(0.284) | -0.330(0.284) | | 0.008(0.001)*** | -0.804(0.384)* | -0.780(0.383)* | | / | LI | |
| Israel | | 0.002(0.001)** | -0.412(0.390) | 0.901(0.390)* | | 0.001(0.001) | -1.008(0.460)* | -0.284(0.460) | | / | / | |
| Italy | | 0.001(0.001) | -0.089(0.308) | 0.324(0.308) | | 0.005(0.001)*** | -0.635(0.457) | -0.005(0.457) | | / | LI | |
| Latvia | | 0.003(0.000)*** | -0.321(0.251) | -0.093(0.251) | | 0.005(0.001)*** | 0.673(0.389) | 0.070(0.389) | | LI | LI | |
| Lithuania | | 0.001(0.001)* | -1.301(0.296)*** | 0.101(0.296) | | 0.001(0.001) | -1.161(0.413)** | 1.280(0.413)** | | QIU | / | |
| Luxembourg | | 0.000(0.001) | -0.971(0.300)** | -0.075(0.300) | | -0.001(0.002) | -0.486(0.423) | -0.969(0.423)* | | / | / | |
| North-Macedonia | | 0.002(0.001) | 0.160(0.251) | 0.228(0.251) | | 0.000(0.001) | 0.707(0.354)* | -0.582(0.354) | | / | / | |
| Malta | | 0.006(0.002)*** | -1.000(0.357)** | 0.383(0.357) | | 0.011(0.002)*** | -0.934(0.431)* | -0.065(0.431) | | LI | LI | |
| Netherlands | | 0.001(0.001) | 0.417(0.238) | -0.060(0.238) | | 0.004(0.001)*** | -0.464(0.377) | -1.266(0.376)*** | | / | C | |
| Norway | | 0.001(0.000)* | 0.266(0.244) | -0.313(0.244) | | 0.002(0.001)** | -0.318(0.357) | -0.297(0.357) | | / | / | |
| Poland | | 0.002(0.000)*** | -0.931(0.271)*** | -0.863(0.271)** | | 0.005(0.001)*** | -0.993(0.385)** | -1.199(0.385)** | | QIU | LI | |
| Portugal | | 0.000(0.001) | 0.375(0.233) | -0.364(0.233) | | 0.001(0.001) | 1.115(0.373)** | 0.092(0.373) | | / | / | |
| Romania | | 0.000(0.001) | -0.524(0.316) | 0.227(0.316) | | -0.003(0.002) | -0.768(0.428) | -0.628(0.428) | | / | / | |
| Russia | | 0.003(0.001)*** | -1.171(0.303)*** | -0.193(0.303) | | 0.003(0.001)*** | -1.039(0.384)** | -0.461(0.384) | | QIU | LI | |
| Scotland | | 0.001(0.000)** | 0.426(0.271) | -0.007(0.271) | | 0.004(0.001)*** | 1.735(0.395)*** | 0.172(0.395) | | / | QU | |
| Slovakia | | 0.003(0.001)*** | -0.613(0.283)* | -0.349(0.283) | | 0.004(0.001)*** | -0.009(0.390) | -0.172(0.390) | | LI | LI | |
| Slovenia | | 0.000(0.001) | -0.151(0.227) | 0.147(0.227) | | 0.006(0.001)*** | 0.861(0.334)** | -1.035(0.334)** | | / | LI | |
| Spain | | -0.002(0.001)** | -0.255(0.279) | -0.790(0.279)** | | -0.004(0.001)*** | 0.390(0.404) | -1.116(0.404)** | | / | LD | |
| Sweden | | 0.001(0.001)* | -0.328(0.291) | 0.320(0.291) | | 0.004(0.001)*** | -0.403(0.419) | 0.602(0.419) | | / | LI | |
| Switzerland | | 0.001(0.000) | -0.276(0.235) | -0.108(0.235) | | 0.003(0.001)*** | -0.102(0.367) | -0.086(0.367) | | / | LI | |
| Ukraine | | 0.000(0.001) | -0.477(0.272) | 1.025(0.272)*** | | 0.000(0.001) | 0.344(0.399) | 1.170(0.398)** | | C | / | |
| USA | | -0.004(0.001)** | 0.385(0.331) | 0.218(0.331) | | -0.002(0.002) | -0.901(0.441)* | -1.140(0.440)** | | / | / | |
| Wales | | 0.000(0.000) | 0.639(0.280)* | 0.421(0.280) | | 0.004(0.001)*** | 0.558(0.405) | 0.657(0.405) | | / | LI | |

Notes. Estimates(Standard error); P<0.001 = ***; P<0.01=**; P<0.05=*; LI = linear increase; LD= linear decrease; QU = quadratic U-shaped; QIU = quadratic inverted U-shaped; C = cubic
